# Rivers in transition: Local perceptions of a Swedish dam removal

**DOI:** 10.1007/s13280-026-02396-w

**Published:** 2026-04-24

**Authors:** Emma Gudmundson, Sanna Stålhammar, Henrik Thorén, John J. Piccolo, Johannes Persson, P. Anders Nilsson, Gary Brierley, Anna Scaini

**Affiliations:** 1 Department of Physical Geography, Stockholm University, 10691 Stockholm, Sweden; 2 Department of Landscape Architecture, Planning and Management, Swedish University of Agricultural Sciences, Alnarp, Box 190, 234 22 Lomma, Sweden; 3 Department of Philosophy, Lund University, 221 00 Lund, Sweden; 4 River Ecology and Management Research Group (RivEM), Department of Environmental and Life Sciences, Karlstad University, Karlstad, Sweden; 5 Department of Biology – Aquatic Ecology, Lund University, Ecology Building, Naturvetarvägen 6A, 22362 Lund, Sweden; 6 School of Environment, University of Auckland (Waipapa Taumata Rau), Auckland (Tamaki Makaurau), Aotearoa New Zealand; 7 Bolin Centre for Climate Research, Stockholm University, 10691 Stockholm, Sweden

**Keywords:** Dam removal, Human–river relations, Hydropower, Mixed-methods, River restoration, Sociocultural values

## Abstract

Effective and locally supported river restoration requires attention to the social dimensions of rivers. This paper examines local perceptions of hydropower and restoration in the river Rönne å, an early case under Sweden’s National Plan for Modern Environmental Conditions for Hydropower. A mixed-methods approach, including a questionnaire and qualitative interviews, explores how inhabitants relate to their river and view the removal of three low-production dams, offering one of the most detailed assessments of stakeholder values in a Swedish hydropower river. The findings reveal important tensions between energy production and ecological restoration: While hydropower retains cultural legitimacy, support declines when ecological costs outweigh energy benefits. The river holds strong recreational, cultural, and symbolic meanings, embedding dam removals in broader sociocultural contexts rather than solely technical or ecological. These findings highlight the importance of participatory efforts and governance that align ecological goals with the diverse ways people relate to rivers.

**Supplementary Information:**

The online version contains supplementary material available at https://doi.org/10.1007/s13280-026-02396-w.

## Introduction

Society faces the twin existential crises of anthropogenic climate change and biodiversity loss. Reducing dependence on fossil fuel is essential to addressing these challenges—but the transition must be both socially and ecologically just (Pörtner et al. 2023; Washington et al. 2024). Sweden has pledged to become fossil-free within one generation (Fossil Free Sweden, n.d.), a goal that requires continued reliance on renewable energy sources such as hydropower. Hydropower supplies around half of Sweden’s electricity (Swedish Energy Agency 2023), but has also fragmented river connectivity and caused major biodiversity impacts worldwide (He et al. 2024). In 2022, the Swedish Government initiated one of the first coordinated nationwide hydropower relicensing programs (the National Plan for Modern Environmental Conditions for Hydropower, NAP) to improve ecological status in regulated rivers. The NAP explicitly calls for a collaborative process involving local stakeholders; in this article, we address the question of how local people perceive, relate to, and engage with changing landscapes resulting from one of the earliest large-scale dam-removal projects to be carried out under the NAP.

Internationally, dam removal is an increasingly common response to ecological degradation (Moran et al. 2018; McCaffery et al. 2024), with studies documenting substantial ecological and hydromorphological gains (Giller 2005; Nilsson et al. 2018; Skidmore and Wheaton 2022). Yet social dimensions remain comparatively underexplored (McCaffery et al. 2024). Restoration projects often prioritize measurable ecological or economic outcomes, sidelining local meanings and attachments (Junker et al. 2007; García-Díez et al. 2020; Watz et al. 2021; Cairns et al. 2024), even though communities hold diverse place-based values tied to rivers (Fox et al. 2016) and associated meanings of socio-natures (Castree 2001).

Bridging this gap is crucial, as restoration interventions can profoundly reshape the deep emotional, cultural, and historical relationships people have with rivers (Perry 2009; Fox et al. 2016). Such changes can trigger conflict, as seen in New England, where some residents perceived dam removals as losses of cherished landscapes, with history, identity, and aesthetics deeply influencing resistance to change (Fox et al. 2016). In the Netherlands (Buijs 2009) and Germany (Deffner and Haase 2018), river restorations instead generated positive responses, for example regarding aesthetics and recreation. In Sweden, Lejon et al. (2009) and Jørgensen and Renöfält (2012) showed that opposition often stemmed from the lack of knowledge of dam-removal benefits and from differing stakeholder valuation of environmental services, while Össbo (2023), Inga et al. (2024), and Söderqvist (2025) reported that Sámi communities in northern Sweden are particularly aware of the negative social, cultural, and environmental impacts of hydropower development on their traditional lands. Although differing in conclusions, these studies underscore the importance of considering everyday-life experiences and attachments when defining what constitutes a “good” river environment. Importantly, what is considered “good” depends on for whom, as perceptions of river quality vary between stakeholders and communities, reflecting cultural, social, and personal priorities (Blue 2018). What constitutes a “good” river environment is also dynamic over time, as what is considered healthy or desirable evolves alongside ecological conditions and societal expectations (Gregory and Brierley 2010).

Rivers can be understood as “hybrid objects” that blend natural and social processes, shaping how people perceive, interact with, and care for them (Linton 2022). The notion of place relationships captures these attachments and meanings that people associate with specific locations. Drawing on Yi-Fu Tuan’s (1977) “sense of place”, place relationships encompass material, emotional, social, and historical connections to landscapes (van den Born et al. 2022). Building on this, Anna Tsing (2005) describes place as a “sticky universal”: a concept that travels across contexts worldwide yet always carries local histories and attachments. To conceptualize the varied relationships between humans and nature, various typologies of environmental values have been proposed (e.g., Tadaki et al. 2017). One common distinction is among instrumental values (nature as a resource), intrinsic values (nature’s worth independent of humans), and relational values (meaningful connections through and with nature) (Himes et al. 2024). It is increasingly recognized that to understand and properly include diverse and sociocultural values of ecosystems in decision-making, plural approaches are required (Pascual et al. 2023). Examining sociocultural values of rivers in particular places is an essential but understudied topic that can contribute to more inclusive forms of river governance (Cottet et al. 2022; Garcia et al. 2022).

Place relationships are not static but evolve over time through lived experiences of environmental change and through differing understandings of past conditions (Brierley 2020). The concept of *solastalgia* captures the emotional distress people experience when familiar environments are degraded or transformed (Albrecht et al. 2007). Complementing this, the shifting baseline syndrome describes how the loss of generational knowledge of past environmental conditions can normalize degraded landscapes, influencing how communities assess restoration outcomes (Soga and Gaston 2018). These dynamics also resonate with Claudia Pahl-Wostl’s (2006) work on adaptive water governance. Values of river ecosystems are historically embedded and shape how societies cope with and negotiate change. They also imply that restoration can both strengthen and disrupt place relationships, with consequences for inhabitants’ quality of life (van den Born et al. 2022). Inclusive, participatory approaches are therefore vital, as they can reinforce attachments, foster shared meaning, and encourage stewardship of local ecosystems (Cottet et al. 2022; Dicks 2022).

Restoration decisions are inherently political, shaping whose knowledge and values are prioritized. In Aotearoa New Zealand, for example, Māori worldviews recognize rivers as ancestral, spiritual beings with agency, where human well-being is inseparable from river health (Hikuroa et al. 2022). Such perspectives align with calls for more-than-human sensitivity in geography, which stress the need to acknowledge rivers as active participants in socioecological systems rather than passive resources (Sharp et al. 2022). Pluralistic perspectives highlight that river restoration is not only about ecological repair but also about rebuilding human–river relationships, recognizing rivers’ intrinsic value and integrating ecological, cultural, and social well-being into governance (Cottet et al. 2022; Hikuroa et al. 2022). By legitimizing diverse perspectives, they support more inclusive governance and position communities as active agents of change (Hikuroa et al. 2022).

In Sweden, research and practice have rarely examined how inhabitants relate to their local river and perceive restoration through dam removal (Lejon et al. 2009; Jørgensen and Renöfält 2012), jeopardizing public support and prospects for restoration success (Lejon et al. 2009; Jørgensen and Renöfält 2012; Germaine et al. 2022; Pott et al. 2025). Yet, as Sweden pursues the dual commitments of a fossil-free energy transition and nationwide relicensing through the NAP, understanding local stakeholder perceptions toward hydropower remediation is required to balance competing sustainability goals such as clean energy, biodiversity, and social justice.

This study investigates local relationships with a river and perceptions of hydropower and restoration through a case study of the river Rönne å, southern Sweden. The river Rönne å is one of the first proposed dam-removal sites under the NAP and is a typical river drainage for southern Sweden, with three main-stem dams that have historically interrupted fish migration and ecological processes. Using a mixed-methods approach combining questionnaire and interview data, the study captures both spatially explicit patterns and narrative accounts of river relationships and restoration perceptions for this first planned dam removal, which should serve as a model for future studies.

## Materials and methods

### Study area

**Fig. 1:**
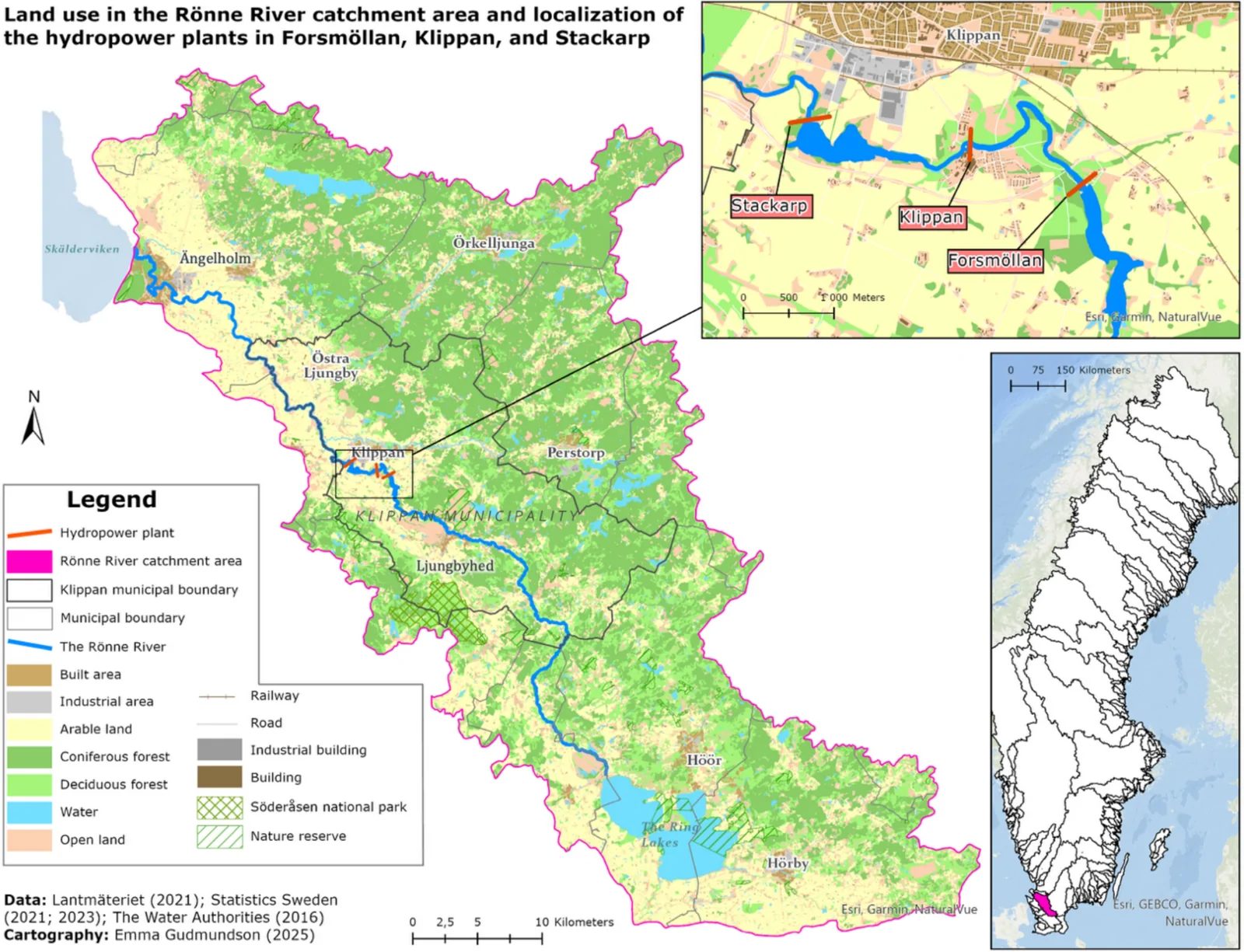
Map of the river Rönne å catchment in Sweden, illustrating land use, catchment and municipal boundaries, and the locations of the three hydropower plants involved in the restoration project

The river Rönne å is a low-relief glaciated catchment originating in the Lake Ringsjön in central Scania and flowing 83 km into the Kattegat in northwestern Scania, southernmost Sweden. Its 1897 km^2^ catchment spans six municipalities, dominated by forests and arable land. The river passes through Klippan municipality, where bedrock formations create dramatic rapids and the river reaches its steepest natural gradient (Klippan Municipality 2025a). The only hydropower plants on the main channel are located here, just south of Klippan town by Forsmöllan, Klippan (the paper mill), and Stackarp (Fig. 1; Figure S7).

Klippan is a sparsely populated municipality, with 17 700 inhabitants spread across 374 km^2^. About three quarters of the population live in urban areas and one quarter in rural settlements (Statistics Sweden 2024). Klippan town, the largest settlement, has about 8000 residents (Klippan Municipality 2025b). The municipality inhabitants have an average age of 43.6 years, slightly above the national mean (Statistics Sweden 2024).

The region has a long industrial history. Forsmöllan was documented as an industrial site as early as 1365 and has operated as a hydropower plant since the late nineteenth century. The Klippan paper mill, completed in 1639, is Sweden’s oldest still-operating paper mill, while Stackarp has hosted water-related industries since the seventeenth century. The river was further impounded in the mid-twentieth century, with the construction of the Stackarp dam in the 1950s and expansions at Forsmöllan in the 1980s (County Administrative Board of Scania 2018), reflecting a nationwide period of hydropower development in which socioeconomic benefits of hydropower were prioritized over environmental concerns (Lindström and Ruud 2017). Together, these three plants produced an average of 9.7 GWh annually, roughly the electricity use of 485 detached houses or the output of two mid-sized wind turbines in Scania, though they offered no regulatory capacity (Klippan Municipality 2025a).

Regionally, the river Rönne å is classified as a “water of national importance” for nature conservation and recreation, with several Natura 2000 areas and opportunities for angling, birdwatching, canoeing, and hiking (Klippan Municipality 2022). The river supports species such as salmon, eel, and endangered mussel populations, yet its ecological status remains only “moderate” under the EU Water Framework Directive (WFD) due to eutrophication, damming, and channel modification (LIFE CONNECTS n.d.-b). Remaining dams create slow-flowing and deeper river sections, producing lake-like habitats with negative effect on water flow and river connectivity for dispersing and migration species.

Hydropower on the river Rönne å main channel is now at a turning point. Following a 2016 court ruling requiring improved fish passage, Klippan Municipality acquired Forsmöllan, Klippan, and Stackarp in 2019 and ceased electricity production. Pending a court decision expected in 2026, the Municipality plans to remove the dams and dismantle the hydropower plants, with the dual aim of restoring hydromorphological processes to support riverine species and climate adaptation, while also generating socioeconomic benefits through recreation and ecotourism (Klippan Municipality 2025a). The restoration is part of the LIFE CONNECTS initiative targeting seven south-Swedish rivers and involves collaboration among national, regional, and local actors (LIFE CONNECTS n.d.-a). Dam removal would restore free flow to the river’s original course, reconnecting 100 km of habitat and enabling unimpeded fish migration and sediment transportation. Efforts will also include placing boulders and spawning gravel to counteract the historical clearing and straightening of the river (LIFE CONNECTS n.d.-b). The restoration will significantly alter a landscape shaped by centuries of hydropower infrastructure (Klippan Municipality 2025a), including the disappearance of dam reservoirs, the return of a free-flowing river, and partial dismantling of hydropower facilities (Figure S8).

The river Rönne å case illustrates broader Swedish challenges, where extensive hydropower infrastructure has created tensions between renewable energy production, ecological restoration, and cultural values (Swedish Committee on Civil Affairs 2022). Although the WFD has been incorporated into the Swedish Environmental Code since 2004 (SwAM 2018), its environmental quality standards remain unmet (VISS 2025). Hydropower is the single largest pressure on Swedish rivers, yet only about 10% of roughly 2000 plants generate around 90% of total output (~ 67 TWh/year) and nearly all regulatory capacity (SwAM, 2019), highlighting opportunities to modernize high-production plants while decommissioning many low-production ones. To address this, the Swedish Government initiated the NAP, under which most hydropower plants will be reviewed over the next 20 years and required to either upgrade to meet current environmental standards or be dismantled (Swedish Committee on Civil Affairs 2022; SFS 1998:808, chapter 11, §§27–28, updated by SFS 2018:1407).

### Data collection

#### Questionnaire

A questionnaire was used to collect quantitative data on local inhabitants’ relationships with the river Rönne å and perceptions of the restoration project, enabling identification of correlations and trends (for more detailed information, see Gudmundson 2025). Designed for multiple river studies, the questionnaire began with a filter question to tailor river-specific questions. The 12 questions analyzed in this study and the full questionnaire is in Appendix S2. Available in Swedish and English, it included an introduction explaining the project and data management policies, with mandatory consent before participation (Appendix S4). The questionnaire was primarily closed-ended and presented vertically, commonly used to allow a wider sample to participate and to facilitate statistical analysis (Bryman 2016; Rahi 2017). The questionnaire incorporated a Public Participation Geographic Information System (PPGIS) exercise, allowing respondents to mark their favorite spot along the river on a digital map. PPGIS offers tools for incorporating local values and priorities in environmental decision-making and is increasingly used internationally to spatially map place-based values (Garcia-Martin et al. 2017; Jaligot et al. 2019; Scaini et al. 2021, 2022), but it is seldom integrated within mixed-method approaches. A follow-up open-ended question invited brief justifications (limited to 20 characters to limit the risk of sharing sensitive information). Additionally, landscape change images from Klippan Municipality’s website (Figure S8) prompted opinions on the potential restoration outcomes.

The questionnaire was piloted with participants familiar with the project and Klippan municipality at varying levels and adjusted based on their feedback to improve response rates and demographic relevance (Bryman 2016). The questionnaire was open from September 25, 2024, to March 3, 2025. The sampling unit comprised inhabitants near the river Rönne å, particularly in Klippan municipality. Distribution used two sampling strategies, based on the idea that various sampling strategies allow for flexibility and diversification of the sample (Patton 1990). First, online distribution was used, relying on convenience sampling (Bryman 2016). A self-completion web questionnaire was distributed through online forums, social media groups (e.g., Facebook), and interest organizations. Due to the nature of convenience sampling, response rates and population representativeness remain unknown (Rahi 2017). Second, postal distribution was used, relying on simple random sampling. 5000 postcards containing a QR code (Appendix S3) linking to the questionnaire were mailed to randomly selected addresses in Klippan municipality. The total sample size is 304 respondents; however, the exact contribution of each sampling strategy is unknown. Based on mailed postcards, the maximum response rate was approximately 6%.

#### Qualitative interviews

Semi-structured interviews were conducted to explore the relationships local inhabitants have with the river Rönne å and their perceptions of the restoration project. An opportunistic sampling strategy was used, inviting questionnaire respondents to volunteer, to further understand the respondent’s narratives and choices (Brown et al. 2012). Nine individuals expressed interest, and the first eight to respond were interviewed. Two additional opinions were received via email from respondents who declined formal interviews.

Interview questions were developed based on preliminary questionnaire results. Interviewees received an information sheet (Appendix S4) and consent form (Appendix S5) one week prior but were not given the interview guide (Appendix S6) in advance to ensure spontaneity. Most interviews integrated the participatory mapping and illustration exercises from the questionnaire, allowing observation of the interviewees’ interactions with these materials (e.g., Figure S8). In the interviews, the participating researcher played an active role in shaping the conversations. Six interviews were conducted face-to-face, two held digitally, and one was a joint interview requested by the participants; the rest were one-on-one. Interviews lasted between 30 and 80 min. All sessions were audio-recorded and transcribed nearly verbatim, omitting only filler words and incomplete sentences. Interviews were conducted in Swedish, with findings carefully translated into English by the authors, who are fluent in English and Swedish. Interviewees were pseudonymized following ethical requirements (Appendix S4).

**Table 1:** Pseudonyms used for interviewees and questionnaire respondents

Interview	Respondent	Flexibility	Pseudonym
1	1	Semi-structured	L1
2	1	Semi-structured	L2
3	1	Semi-structured	L3
4	1	Semi-structured	L4
4	2	Semi-structured	L5
5	1	Semi-structured	L6
6	1	Semi-structured	L7
7	1	Semi-structured	L8
8	1	Email	R1
9	1	Email	R2

### Data analysis

#### Questionnaire data analysis

All responses from participants aged 18 and older were included in the analysis. Quantitative analyses were primarily conducted using Microsoft Excel (version 16.94). The questionnaire included interval, ordinal, and nominal variables, which were analyzed using univariate statistical methods. Results were visualized with bar charts, pie charts, and histograms to illustrate response distributions. Some responses were summarized descriptively across relevant respondent groups (Figure S9–Figure S13), while most qualitative responses were numerically coded (e.g., assigning -5 for “Very negative”). For Likert scale and slider questions, responses were grouped into positive and negative categories. Neutral responses were split evenly between these groups. These results were displayed as percentages of total responses (Figs. 4, 5).

Two georeferenced maps of respondents’ favorite places were created in ArcGIS Pro using spatial data extracted via Survey123 and exported as a shapefile. First, a kernel density heatmap visualized the continuous distribution of favorite locations, applying a fixed radius of 5 map units and equal weighting for all points to highlight clustering (Fig. 3). Second, a point density map calculated the number of points within a 1000-m radius neighborhood around each 100-m raster cell, also with equal weighting. The output was symbolized into five manually defined classes, representing spatial frequency across the river Rönne å catchment (Figure S14).

Open-ended responses (Q12) were analyzed by identifying recurring words, which were carefully translated from Swedish to English and grouped into keyword categories. The 15 most common groups were visualized using a word cloud and bar chart (Figure S15).

#### Qualitative interview data analysis

Transcribed interview responses were organized into tables by question to facilitate thematic analysis. This structure aided in identifying recurring themes within the qualitative data. Theme development focused on recognizing patterns, similarities and differences, theory-relevant content, and metaphors or analogies (Braun and Clarke 2022). The thematic analysis approach incorporated elements of grounded theory, grounding insights in the empirical data and employing an iterative process where data collection and analysis informed each other (Corbin and Strauss 2014). Focused coding prioritized the most salient and revealing themes (Charmaz 2006). The thematic framework—comprising two themes—was applied to both the qualitative and quantitative data sets for consistency.

## Results

### Questionnaire results

#### Participants’ relationships with the river Rönne å

A total of 304 respondents completed the questionnaire. Participants ranged in age from 18 to 95 years, with the 55–65 age group being the most common. Of these, 62% (189 participants) identified as men, 35% (107) as women, and 1% (3) as other genders, while 2% (5) preferred not to respond. Approximately half (49%; 149) lived within 1 km of the river Rönne å, and 89% (270) within 5 km. Respondents reported visiting the river more frequently in summer (29%; 87) than in winter (2%; 7) to engage in activities. Visit frequency varied, with the most common being a few times per month (26%; 80) and a few times per week (22%; 67), while fewer reported a few times per year (16%; 50) or almost daily (13%; 40). 24% (72) had lived in the area for 10 years or less, while 45% (134) had resided there for more than 30 years. 14% (41) reported that their families had lived in the area for less than 5 years, 54% (156) up to 30 years, and 7% (19) more than 80 years (Figure S9–Figure S13).

**Fig. 2:**
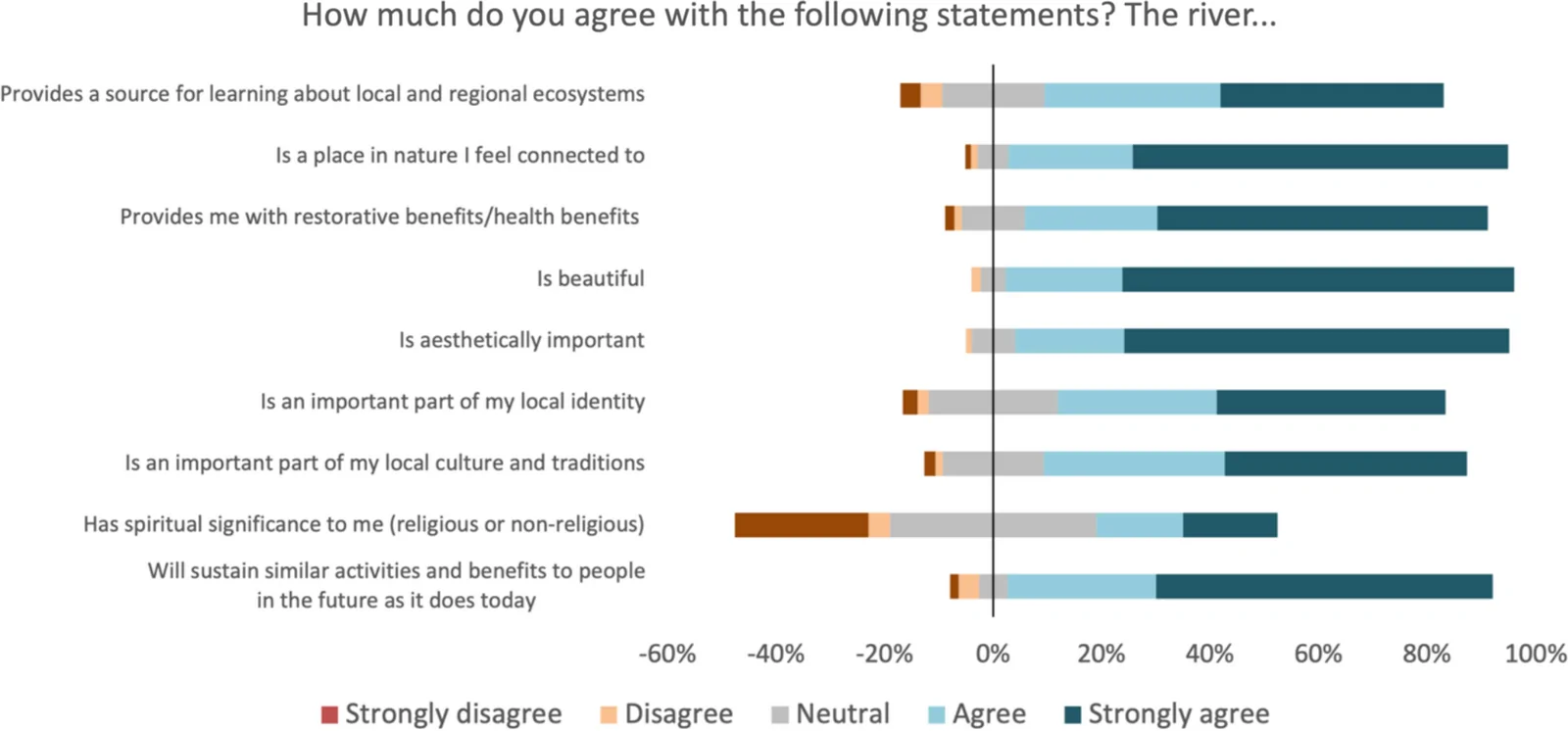
Respondents’ opinions of the river Rönne å's importance for various sociocultural aspects

Most respondents regarded the river Rönne å as significant across several sociocultural aspects. The vast majority felt connected to it (92%; 277), agreed on its beauty (95%; 283) and aesthetics (91%; 275), cultural (78%; 234) and local identity value (71%; 215), contribution to health (85%; 255) and learning (73%; 222), and continued future importance (89%; 268). Responses were more divided regarding the river’s spiritual significance, with similar agreement (33%; 100) and disagreement (29%; 86) (Fig. 2).

**Fig. 3:**
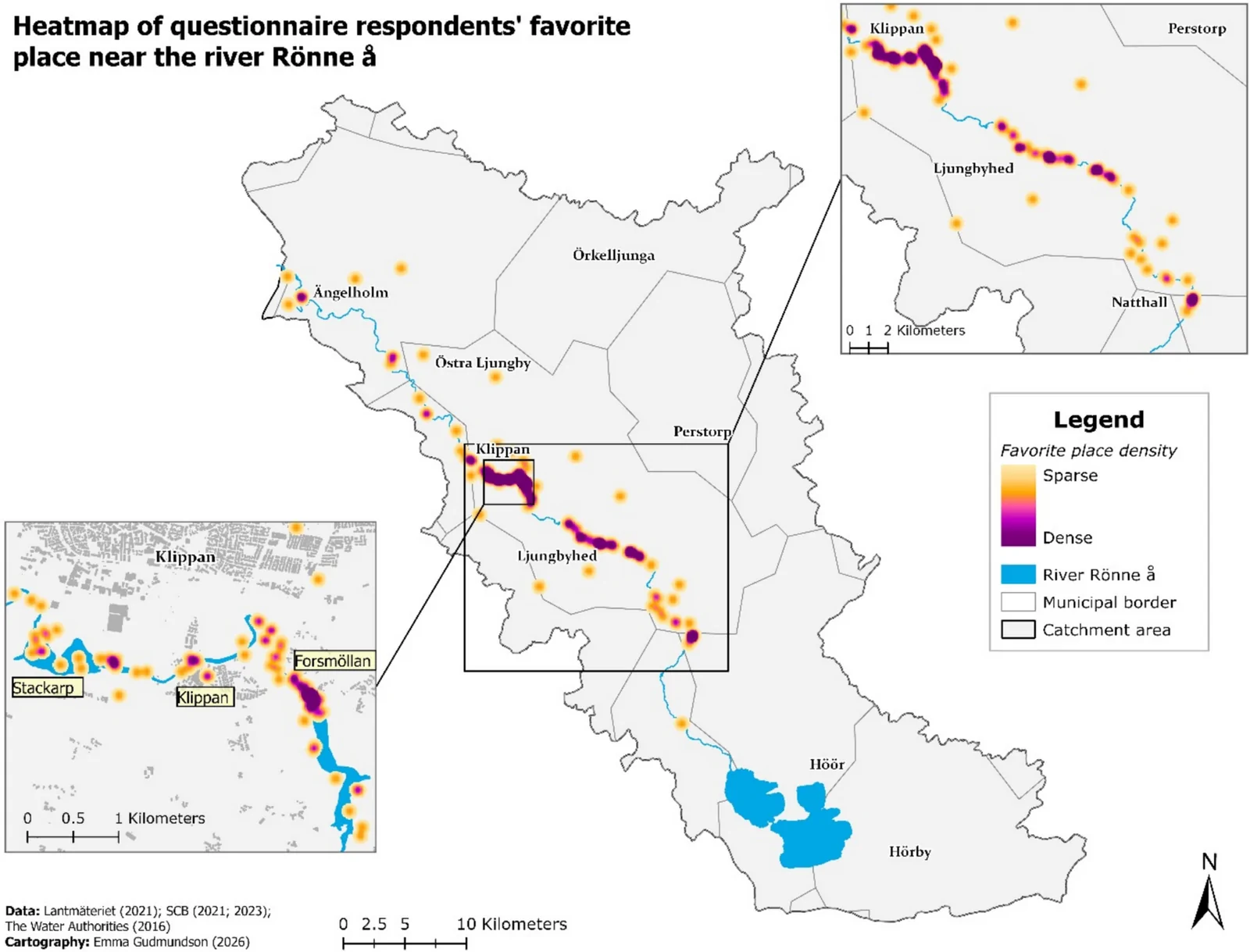
Heatmap showing the spatial distribution of questionnaire respondents’ favorite places along the river Rönne å

Three areas near the river emerged as favorite places, all within Klippan municipality: the stretch south of Klippan town near the three hydropower plants, a section east of Ljungbyhed, and the area around Natthall in the municipality’s southeastern corner (Fig. 3). Forsmöllan exhibited the highest concentration of favorite places (20,1 points/km^2^) (Figure S14). The most frequent keyword groups from free-text responses were ‘Beautiful’ (24%; 71), ‘Close to home’ (10%; 30), and ‘Peaceful’ (9%; 28) (Figure S15).

#### Perceptions of hydropower and the restoration

**Fig. 4:**
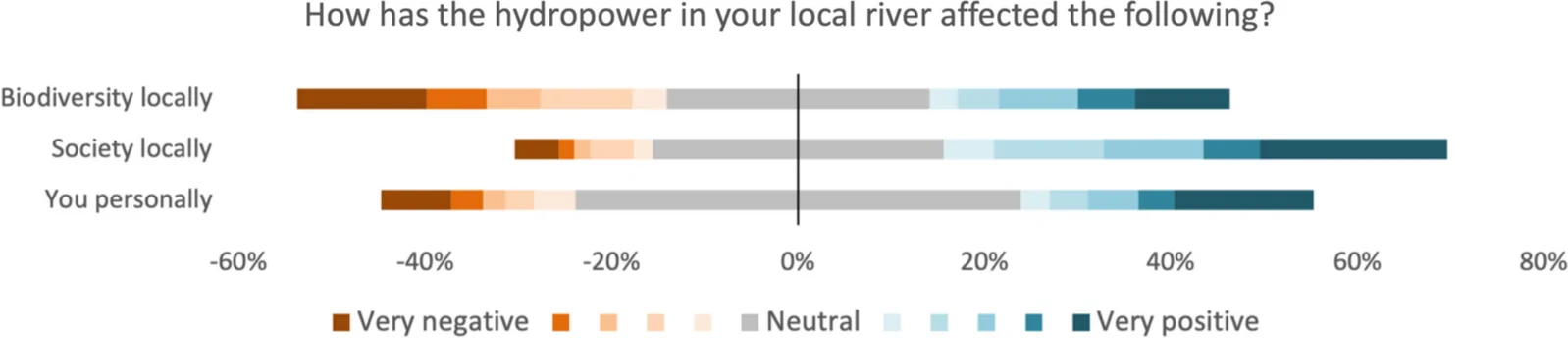
Respondents’ opinions on how hydropower in the river Rönne å has affected local biodiversity and society, and themselves personally

A slightly larger proportion of respondents perceived hydropower to have negatively impacted local biodiversity (38%; 117), while most viewed its effects on local society as positive (53%; 161). On a personal level, more respondents felt positively affected (30%; 92), though a substantial number remained neutral (46%; 140) (Fig. 4). The majority believed current hydropower and damming have positively influenced industrial use (58%; 177), recreational activities (64%; 196), tourism (60%; 181), residents’ everyday well-being (60%; 183), the natural environment (63%; 192), biodiversity (59%; 180), livestock (51%; 156), and fossil-free energy (54%; 165). Fewer respondents reported negative impacts on these aspects. However, more respondents viewed commercial fishing as negatively affected. Neutral responses dominated issues such as infrastructure (44%; 134), water pollution (53%; 161), hydromorphology (44%; 134), electricity prices (53%; 160), and climate change (55%; 167), with positive and negative opinions more evenly distributed (Figure S16).

**Fig. 5:**
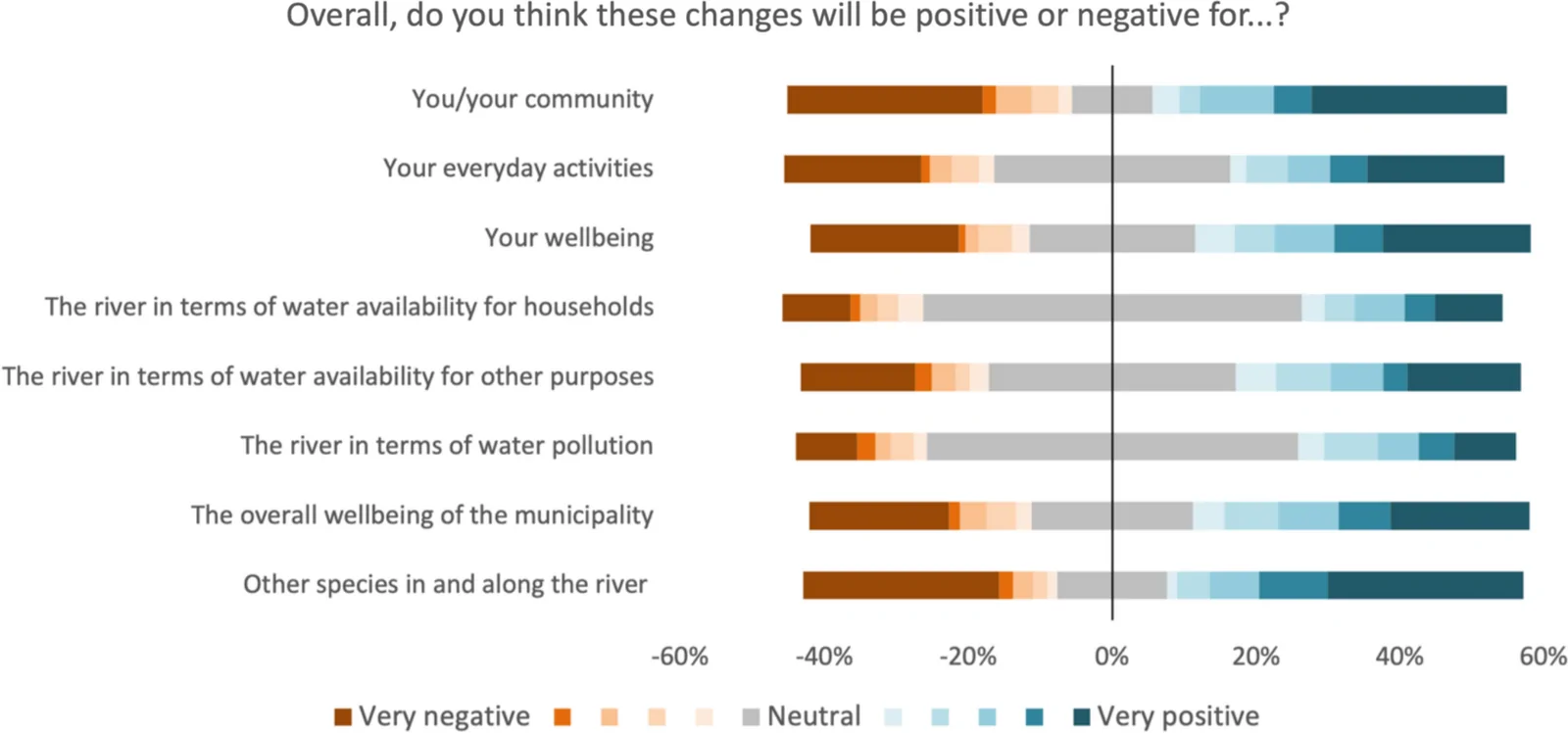
Respondents’ opinions on the anticipated effects of the river Rönne å restoration on various aspects

Regarding the anticipated effects of the river Rönne å restoration on various personal, societal, and environmental aspects, respondents expressed a mix of positive and negative views, with a moderate positive leaning (~ 60/40-ratio). Strong opinions—both positive and negative—were common about the project’s expected impact on individuals, communities, and riverine species. In contrast, respondents showed greater uncertainty or neutrality about impacts on daily activities (35%; 102), household water availability (50%; 151), water uses (34%; 104), and water pollution (48%; 145) (Fig. 5).

### Interview results

#### Participants’ relationships with the river Rönne å

The eight interviewees ranged from 30 to 83 years old, comprising six men and two women. Most lived in Klippan municipality, with one residing in Ängelholm municipality. Many had lived within walking distance of the river Rönne å. All reported visiting the river at least a few times per year for recreation and described strong personal connections to it. Interviewees agreed that repeated visits and shared memories have deepened these relationships over time (see more in Appendix S8).

Some interviewees did not have a specific favorite place along the river but appreciated its overall aesthetics and recreational value. Others cherished spots tied to memories and emotions, especially those easily accessible from home. One interviewee highlighted sites that helped them symbolize life moments, for instance, describing a riverbank that occasionally floods: “*Just like life sometimes overflows with things that you must do. […] it also becomes an image, that this is what it can look like, it can be flooded, but it then goes back to the normal course*” (L2). Beyond the tangible, this interviewee also emphasized rivers’ roles as connectors of landscapes, forming natural networks like roads or nervous systems (see more in Appendix S8).

One questionnaire respondent echoed this perspective, emphasizing enjoyment derived from observing nature rather than actively using its resources, and highlighting the importance of recognizing the rights of non-human species. They suggested that humans might not automatically have rights to all parts of the river and that human interactions—both before and after restoration—shape the well-being of the entire river ecosystem (R2). Similarly, an interviewee argued that the river itself should be given a voice in decision-making, stressing that municipalities and their partners ought to act as its representatives: “*That the river has a say, that it cannot just be used for hydropower*” (L2).

#### Perceptions of hydropower and the restoration

Most interviewees expressed support for the restoration project, while others were critical. Interviewees generally spoke optimistically about the ecological goals, highlighting benefits for wildlife in and around the river. Reflecting on the trade-offs, one interviewee noted that “*those power plants did not produce that much electricity, but hopefully they can provide greater benefits for nature”,* emphasizing that *“for biodiversity, […] it is something I think is very important, because in the long run, our lives also depend on it*” (L6). Supporters valued knowing that the ecosystem would function more effectively, even if they could not always describe the exact impacts and acknowledged that biodiversity benefits would not always be directly perceptible but still appreciated the long-term ecological focus of removing the dams.

A recurring critical perspective was that closing functioning hydropower plants is an unwise environmental and political choice. However, most interviewees agreed on the three hydropower plants’ insignificant effect on the electricity system. One interviewee suggested that much local skepticism reflects a general resistance to change and mistrust of political institutions rather than specific objections to the restoration itself (see more in Appendix S8). Drawing parallels with previous local projects, the interviewee predicted that acceptance would grow once the project’s benefits become visible:

*Inside Klippan, where they have restored the leather factory [...] We who grew up in Klippan, the leather factory was central. I do not think a single person is sad that it is gone, and they have tried to restore a piece of land and make it available to the citizens. I think the same thing will happen with the power plants; in time, people will think it is good.* (L8)

Interviewees expressed mixed views on anticipated landscape changes (Figure S8, see more in Appendix S8). Some valued the current dam landscapes, appreciating tranquil areas like Forsmöllan, with one questionnaire respondent noting that the existing landscape has shaped the upbringing of at least three generations (R1). Others described the dams as more of a hindrance than an aesthetic feature, recalling canoeing through the Forsmöllan dam, where paddling rattles against submerged trees: “*It looks like a forest underwater, actually. Then you think about it even more, that it was not like that once*” (L3).

The hydropower plants are widely regarded as part of the area’s historical and cultural heritage, having contributed to the local community for centuries. For some, the restoration itself also represents as form of cultural development. One interviewee remarked, “*in a way, this also becomes a cultural landscape because it is we humans who are doing this – the restoration is also by human hands*” (L8). Similarly, another interviewee drew parallels to past modifications of another river, suggesting that altering the landscape can itself preserve history: “*The history remains. It is almost culture because it was redirected. I cannot imagine that it would be negative to just redirect the water, because you can still tell the story about the dam*” (L7).

Several interviewees suggested that raising awareness of the river Rönne å restoration could enhance environmental reflection among locals. One interviewee emphasized that such projects can subtly shape environmental thinking, explaining that initiatives like these may “*train ourselves to think in a certain way*” (L8) and influence broader environmental behavior.

## Discussion

All rivers are products of cumulative impacts, wherein responses to disturbance events (contemporary and legacy effects) play out at the catchment scale (Downs and Piegay 2019). The trajectory of river adjustment is typically nonlinear in terms of both the rate and direction of change—geographical and historical contingencies produce place-based, catchment-specific characteristics (Phillips 2007; Brierley et al. 2013). In turn, biophysical conditions and societal perceptions of a “healthy” river are shaped by sociocultural values and by the policy, legal, governance and institutional frameworks that assert particular values upon rivers (Blue 2018; Brierley 2020). These dynamics unfold in distinctive ways in the low-relief, glacially denuded landscapes of southern Sweden, where rivers have experienced centuries of anthropogenic modification.

Although dam removal is a global phenomenon, its extent and implementation vary considerably across regions (Rudberg et al. 2015). Careful case study research is therefore needed to understand the deeply contextual social and environmental conditions that shape each instance. The mixed perspectives on dam removal for the river Rönne å outlined in this study reflect long-term societal relationships with the river. As these relationships continue to evolve, they will in turn shape future outcomes for the river and its management.

This study provides insight into how local inhabitants relate to their river and perceive hydropower and restoration initiatives. While ecological and technical perspectives have long dominated both Swedish and international river restoration research, sociocultural insights at this breadth remain rare. Given Sweden’s dual objectives of maintaining renewal energy production while restoring biodiversity, incorporating local knowledge and values is essential for project success. The findings demonstrate both local support for restoration and contested cultural attachments to hydropower, highlighting the value of a mixed-methods approach for capturing diverse perspectives on river futures.

### Diverse relationships with the river Rönne å

Questionnaire respondents and interviewees were predominantly residents who live near the river Rönne å, visit it frequently, and have long-standing ties to the area. This familiarity has fostered strong personal and emotional connections with the river, such that attachments express multiple roles of the river in people’s lives and experiences (Junker et al. 2007; Germaine et al. 2022; Cairns et al. 2024). Participants highlighted the importance of aesthetics, local heritage, cultural identity, learning opportunities, and personal memories, while others described the river as a symbol and metaphor. Perceptions of the river thus reflect instrumental, relational values, and in some cases, intrinsic values (Tadaki et al. 2017). Favorite places were identified along the entire main channel, reflecting its aesthetic quality, accessibility, and positive experiences associated with spending time there. The notable concentration of favorite places near the dams indicates that inhabitants already attach special significance to the area targeted for restoration. As sociocultural relations to rivers and place relationships are dynamic across space and time (Brierley 2020), restoration projects may either reinforce or unsettle evolving connections, affecting inhabitants’ quality of life (van den Born et al. 2022).

Importantly, these attachments are not contingent on hydropower production. The river itself was experienced as a meaningful landscape-shaping local identity, with respondents stressing human dependence on the river’s health and noting its role as a sustained provider of future benefits. One participant emphasized the river’s value independent of human use, highlighting that human interactions—past and future—shape ecosystem well-being. Another perspective emphasized that the river itself should be given a voice in decision-making. Such assertions of more-than-human sensitivity (Sharp et al. 2022) see rivers as active participants in sustaining life (human and non-human) rather than as passive backdrops for human activity (Salmond et al. 2019). A more-than-human sensibility also recognizes rivers as dynamic actors whose futures depend not just on management but on cultivating enduring, adaptive, and ecocentric human-nature relationships (Brierley 2020; Washington et al. 2024). Plural and diverse values thus shape river realities (Hikuroa et al. 2022; Pascual et al. 2023).

### Hydropower and restoration perceptions

The study shows that inhabitants near the river Rönne å generally accept hydropower and associate it with positive values, even while recognizing ecological costs, indicating cultural legitimacy. This acceptance likely reflects Sweden’s long history of living with hydropower, its role in the country’s climate-positive narrative, and perceived necessity for national energy security (Lindström and Ruud 2017). Hydropower facilities, including the low-production dams in the river Rönne å, are deeply embedded in the cultural heritage of the region and is often perceived as part of the familiar landscape rather than a disruptive force (Söderqvist 2025).

At the same time, perceptions of hydropower’s societal and ecological impacts were mixed, and most participants expressed positive expectations for dam removal in the river Rönne å. These views appear closely linked to deeper socionatural relationships with the river that shape how environmental change is perceived and interpreted. Interview responses draw attention to key constructs of socionatural relationships, such as the shifting baseline syndrome (Doga and Gaston 2018) and solastalgia (Albrecht et al. 2007), expressing the distress people feel when their environments are degraded or transformed in ways that disrupt familiar attachments. These perspectives point to the importance of social learning processes in which historically embedded values mediate societal responses to environmental change which shape how communities cope, negotiate, and gradually learn to adapt to environmental change (Pahl-Wostl 2006).

Previous studies in Sweden have shown that aesthetic and recreational opportunities associated with dams can become embedded within local senses of place (Lejon et al. 2009; Jørgensen and Renöfält 2012). As described by Tuan (1977), sense of place is always situated and contingent, shaped by time and lived experience. Transformative events such as dam removal can therefore create new and uncertain relationships with place. Notions of place as a “sticky universal” (Tsing 2005) emphasize how local attachments and meanings are highly particular, yet they resonate with broader debates about how societies relate to place under conditions of ecological change.

Interestingly, some interviewees described restoration as a form of cultural development rather than simply ecological repair. While certain landscape features may disappear, new narratives and relationships with the river could emerge. This underscores the multiplicity of meanings associated with restoration (Cottet et al. 2022; Dicks 2022; Hikuroa et al. 2022; van den Born et al. 2022): for some, it means returning the landscape as close to a near-pristine state; for others, valuing the current landscape; and for still others, integrating restoration as a part of broader cultural change. Restoration therefore emerges not only as an ecological or hydromorphological intervention, but also as a social and cultural process (Gregory and Brierley 2010; Blue 2018). The emergence of new narratives and attachments around the river Rönne å highlights the evolving nature of human–river relationships, suggesting that restoration processes may generate as much cultural transformation as ecological repair. These perspectives also underscore the inherent uncertainties of continually evolving societal relations to rivers and the outcomes that follow (cf., Hillman and Brierley 2008; Brierley et al. 2016). The shifting and situated nature of river meanings has profound implications for future aspirations and governance.

### Policy and practice implications

Profound ethical considerations underpin processes through which legitimacy and local support are incorporated within restoration planning and enactment (Dicks 2022; Pott et al. 2025). Who defines the “value” of the river? This study highlights the primacy of plural perspectives—ecological, economic, cultural, and everyday. Such notions extend beyond technical representations of which values count in river planning and management (McPhearson et al. 2022). Ecological integrity in sympathy with social–ethical relationships with rivers is a profound matter of environmental and epistemic justice (Fox et al. 2017; Dicks 2022; Hikuroa et al. 2022).

The results also identify potential entry points for NAP policy reform. Local support appears to align with the NAP’s central objective of removing facilities that have significant ecological impacts but contribute minimally to national energy production. These findings have broader implications for public debates, where ecological restoration is often framed as incompatible with energy security, thereby prioritizing economic interests over biodiversity (Swedish Energy Agency et al. 2016; Puharinen et al. 2024; Gudmundson 2025). This raises political questions of whose interests such narratives serve—certainly not those of the river itself, nor those relying on its long-term benefits. Integrating sociocultural perspectives is therefore essential not only for local support but also for fulfilling international commitments.

At broader scale, the EU’s Nature Restoration Law (NRL) requires the reconnection of 25 000 km of free-flowing rivers across Europe (Regulation (EU) 2024/1991). The approach developed in this study offers an adaptable framework for incorporating sociocultural perspectives into restoration planning. While the present research focuses on a single river case, future studies could apply similar approaches across Swedish and international rivers to explore the generalizability of these findings.

### Methodological implications

This study demonstrates the value of a mixed-methods approach for investigating local relationships with rivers and perceptions of restoration initiatives. In Sweden, such approaches are particularly relevant as the country has taken a global lead in aiming for fossil-free status within one generation. The next step is ensuring that the energy transition also embraces participatory approaches for environmental initiatives. Integrating participatory and sociocultural perspectives into environmental initiatives may help align local experiences with broader climate, energy, and ecological goals. At the same time, restoration initiatives must remain attentive to situatedness: the NAP begins from a legacy where hydropower has long been framed as synonymous with development, but boundary conditions are now changing (Salmond et al. 2019; Brierley 2020). Locally, restoration may therefore be experienced simultaneously as an opportunity and a threat, reflecting the dynamic ecological, institutional, and social transitions that rivers are currently undergoing.

Several limitations should be acknowledged. The results may not capture the full diversity of community perspectives. For one, environmentally interested individuals may have been more motivated to participate, or possibly only those with strong feelings about the river or the restoration project e.g., reflecting a self-selection bias (Brown et al. 2012). Moreover, since such sentiments should not be assumed to be homogenous among different social groups, e.g., migrant communities, there may be diversity that is not captured by this study. Something similar could probably be said about ways of engaging with particular places, their history and possible futures, and thus the kinds of relations those modes of engagement give rise to. Questionnaire design also limited responses, as no explicit “I don’t know” option was available. Finally, neutral responses for some topics raise interpretative challenges. Such responses may reflect either indifference or limited knowledge about hydropower and restoration effects. Interviewees often struggled to explain exactly how the river ecosystem would function post-restoration, suggesting some impacts are easier to imagine than others.

## Conclusion

This study examined local relationships with the river Rönne å and perceptions of hydropower and restoration during one of the first cases under Sweden’s National Plan for Modern Environmental Conditions for Hydropower (NAP). It explored how inhabitants near the river Rönne å relate to their local river and perceive the removal of three low-production dams. The findings reveal that while hydropower retains cultural legitimacy, this legitimacy weakens when ecological costs outweigh limited energy benefits. At the same time, people attach diverse emotional, cultural, and symbolic meanings to the river, underscoring that dam-removal decisions are not solely technical or ecological, but embedded in broader social and cultural contexts. Participants emphasized reconnecting humans with nature, highlighting dependence on river health, encouraging environmentally conscious behaviors, and advocating for more-than-human governance approaches that give the river a voice. Restoration was also seen as a potential avenue for cultural development, where disappearing structures may be replaced by new narratives, practices, and relationships, reshaping both cultural and ecological landscapes.

These insights have clear implications for policy and practice. Recognizing the diverse and sometimes conflicting sociocultural relationships and values that people hold with rivers is a key component of river governance. Mixed-method approaches targeting local stakeholders provide ways of integrating local values and perceptions into decision-making (Tadaki et al. 2017; Pascual et al. 2023). Rather than perceiving ecological improvement as a threat to development, restoration is a shared investment that generates significant social and environmental benefits. As Sweden advances the NAP and implements the EU Nature Restoration Law (NRL), governance structures need to deliver not only ecological gains but also respect the varied ways people interact with, know, and care for rivers. Without this integration within policy discourse, the potential for sustainable and just energy transitions is likely to remain unrealized.

## Supplementary Information

Below is the link to the electronic supplementary material.

Supplementary file1 (PDF 2397 KB)

## Data Availability

The data that support the findings of this study are available from the authors under reasonable request.
