# Supplementary material for: Rivers in transition: Local perceptions of a Swedish dam removal
Source: Ambio. 2026 Apr 24;55(11):2722–36. doi: 10.1007/s13280-026-02396-w (PMC13661868; doi:10.1007/s13280-026-02396-w)
Supplement: Supplementary file 1 [file 13280_2026_2396_MOESM1_ESM.pdf]

Supplementary Information

Title: Rivers in Transition: Local Perceptions of a Swedish Dam Removal

This Supplementary Information has not been peer reviewed.

Supplementary Information

Appendix S1 – Additional illustrations and photographs of the river Rönne å

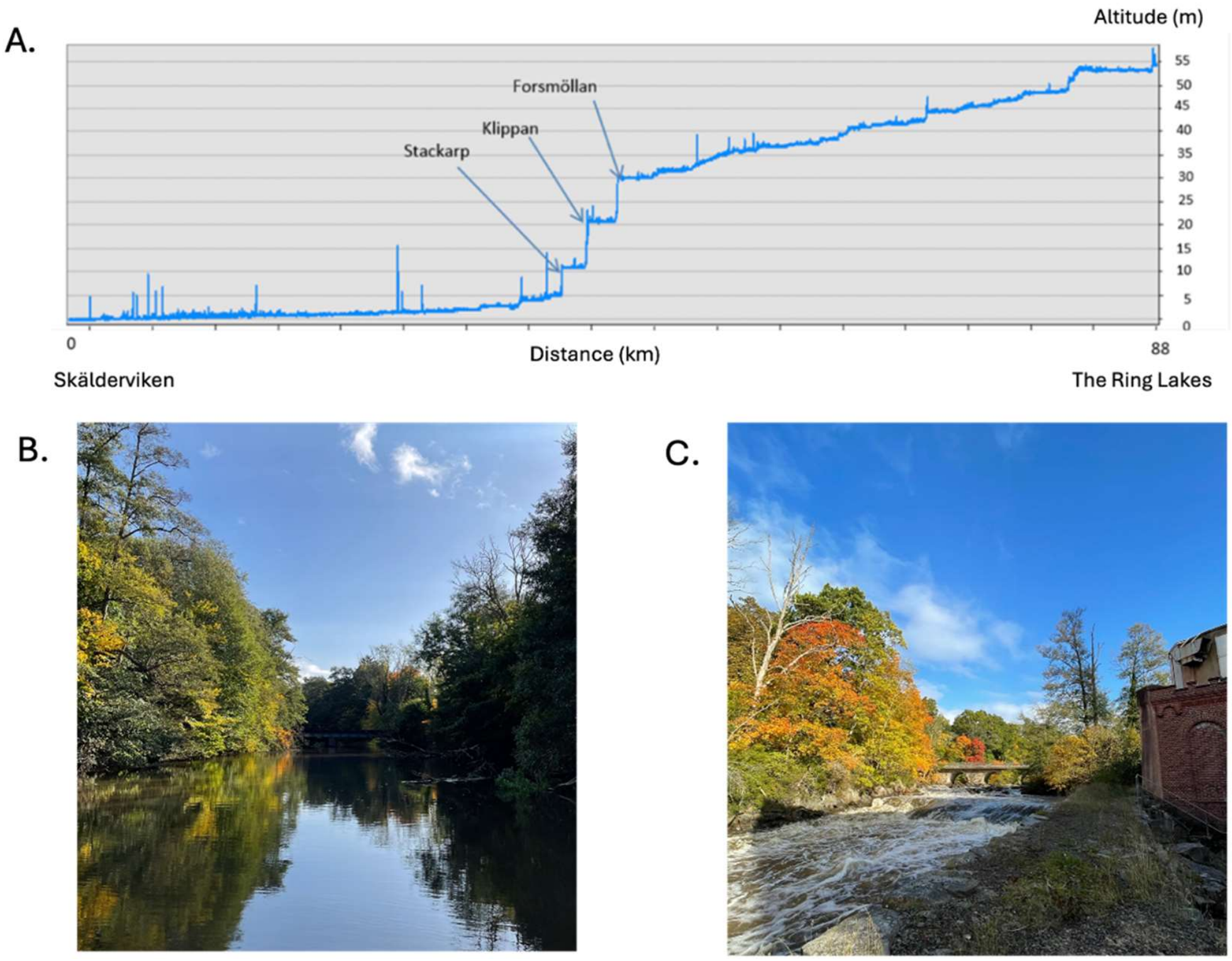

Figure S7: B. Gradient profile of the river Rönne å. Retrieved from Klippan Municipality (2021). C. Upstream view of the river Rönne å near the Klippan power plant (Gudmundson, 2024). D. Upstream view of the river Rönne å with the Klippan power plant to the right (Gudmundson, 2024).

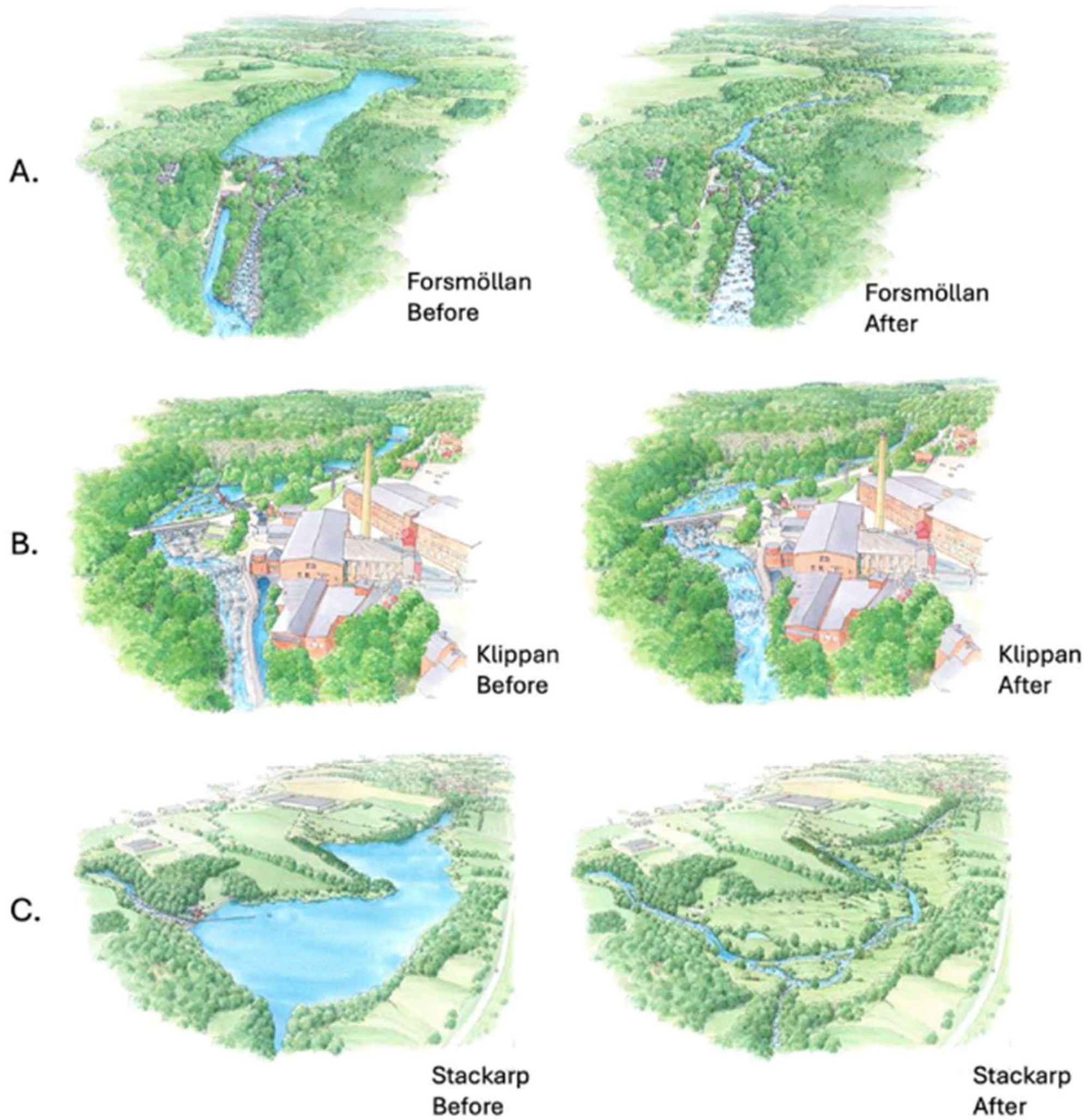

Figure S8: Visualisations of the three hydropower plant sites – A. Forsmöllan, B. Klippan, and C. Stackarp – before and after restoration, used to prompt respondents' opinions on the expected landscape changes. Illustrations by Carina Lindqvist, Naturcentrum, adapted from Klippan Municipality (2023).

Appendix S2 – Questionnaire: Information text for respondents, questions used in this study and the complete questionnaire

Dear reader,

We are a group of scientists from Stockholm University and SLU Alnarp.

Our work aims to understand how local communities perceive their local river and if locals feel a connection with the place they live in and the surrounding nature. Thus, we are distributing this questionnaire to explore your everyday practices by the Rönne å, Luleälven, or Gullspångsälven, and your willingness and participation in the planning and management of the river. Your response will allow us to understand the main activities you perform near the river, and your expectations for the future management of the river.

The questionnaire will take **about 10-15 minutes** to be completed. Your participation in this study is completely voluntary. There are no foreseeable risks associated with this project. However, if you feel uncomfortable answering any questions, you can withdraw from the survey. It is very important for us to know your opinions; there will be no unauthorized access to your responses and the data from this research will only be reported in aggregate form. Your information will be encrypted and will remain confidential. If you have any questions about the survey or any of the procedures, you can contact Anna at anna.scaini@natgeo.su.se.

You can read more about the overall scope of the project here. Please, get in touch if you are interested in our study.

Thanks,

Emma Gudmundson (Stockholm University), Sanna Stålhammar (SLU Alnarp), Anna Scaini (Stockholm University).

Click here to read about how your personal data will be processed

Table S2: Questionnaire questions included in this study. The table displays question ID, question, sentences, scale, responses, and theme.

ID	Question	Sentences	Scale	Responses	Theme
Q1	How close to the river do you live?	Less than 1 km away<br>Between 1 and 5 km away<br>Between 5 and 10 km away<br>More than 10 km away<br>Do not know/prefer not to respond	Single select	304	Profile and Relationship
Q2	How long have you lived in the area?	-	Free text, must be an integer between 0 and 120	301	Profile and Relationship
Q3	How long has your family lived in the area?	-	Free text, must be an integer between 0 and 99999	291	Profile and Relationship
Q5	How often do you visit the river to carry out these activities?	Almost every day<br>A few times per week<br>A few times per month<br>Seasonally, warmer months	Multiple choice	302	Profile and Relationship

ID	Question	Sentences	Scale	Responses	Theme
		Seasonally, colder months			
		A few times per year			
		Do not know/prefer not to respond			
Q6	How much do you agree with the following statements? The river…	Provides a choice for learning about local and regional ecosystems	Likert: 1. Strongly disagree 2. Disagree 3. Neither agree nor disagree 4. Agree 5. Strongly agree	301	Profile and Relationship
		Is a place in nature I feel connected to		300	
		Provides me with restorative benefits/health benefits		299	
		Is beautiful		301	
		Is aesthetically important		301	
		Is an important part of my local identity		300	
		Is an important part of my local culture and traditions		300	
		Has spiritual significance to me (religious or non-religious)		300	
		Will sustain similar activities and benefits to people in the future as it does today		300	
Q7	In your opinion, how has present hydropower and damming in the river affected the following?	Industrial use	Slider: -5= Very negative<br>0= Neither negative nor positive<br>5= Very positive	298	Hydropower and Restoration Opinions
		Recreational activities (e.g., hiking, running, canoeing, fishing, snow fishing)		297	
		Industrial fishing		281	
		Tourism		298	
		Infrastructures		293	
		Residents' everyday well-being		294	
		Ecology/natural environment		300	
		Species richness (biological diversity)		295	
		Water pollution (e.g., temperature changes)		287	
		Hydromorphology (e.g., sediment transport, connectivity, riverbank development)		291	
		Cattle/animals		291	
		Fossil-free energy		293	
		Electricity prices		290	
		Climate (change)		289	
Q9	Overall, do you think these changes will be positive or negative:	For you/your community	Slider: -5= Very negative<br>0= Neither negative nor positive<br>5= Very positive	301	Hydropower and Restoration Opinions
		For your everyday activities		298	
		For your wellbeing		298	
		For the river in terms of water availability for households		291	
		For the river in terms of water availability for other purposes		295	
		For the river in terms of water pollution		292	
		In terms of the overall wellbeing of the municipality		297	
		For other species in and along the river		295	
Q11	What is your favorite place by the river Rönne å?	-	Map indicator	181	Profile and Relationship
Q12	Why is it your favorite place?	-	Free text, must be 20 characters or less	217	Profile and Relationship
Q18	State your opinion about the effects of hydropower	Has the hydropower in your local river had a negative or positive effect on biodiversity locally?	Slider: -5= Very negative<br>0= Neither negative nor positive<br>5= Very positive	295	Hydropower and Restoration Opinions
		Has the hydropower in your local river had a negative or positive effect on society locally?		298	
		Has the hydropower in your local river had a negative or positive effect on you personally?		293	
Q22	How old are you?	-	Free text, must be an integer between 0 and 120	303	Profile and Relationship
Q23	Your gender?	Woman	Multiple choice	303	Profile and Relationship
		Man			

ID	Question	Sentences	Scale	Responses	Theme
		Non-binary<br>Transgender<br>Gender queer/gender fluid<br>Other<br>Prefer not to respond			

Table S3: The complete questionnaire. The table displays question ID, question, sentences, scale type, number of responses, and theme.

ID	Question	Sentences	Scale	Responses	Theme
	Do you consent to participating in the study and to the processing of your personal data in the ways described?	Yes<br>No	Single select	592<br>16	
	Which river is close to you?	Rönne å<br>Luleälven<br>Gullspångsälven<br>None of the above/other	Single select	305<br>198<br>89<br>0	
Q1	How close to the river do you live?	Less than 1 km away<br>Between 1 and 5 km away<br>Between 5 and 10 km away<br>More than 10 km away<br>Do not know/prefer not to respond	Single select	304	Participant Profiles and Relationship with the Rönne River
Q2	How long have you lived in the area? (number of years)	-	Free text, must be an integer between 0 and 120	302	Participant Profiles and Relationship with the Rönne River
Q3	How long has your family lived in the area? (number of years)	-	Free text, must be an integer between 0 and 99999	292	Participant Profiles and Relationship with the Rönne River
Q4	Which types of activities do you engage in/near the river?	Recreational fishing<br>Subsistence fishing<br>Farming/animal grazing<br>Walking/running<br>Skiing<br>Other winter sport<br>Water sports (e.g., canoeing)<br>Snow scooter<br>Tourism<br>Recreational nature activities (e.g., hiking, camping)<br>Aesthetics (e.g., photography, filmmaking, painting, visual arts)<br>Social or cultural activities (e.g., museums)<br>Water use (e.g., irrigation)<br>Hydropower<br>Other industry<br>Other<br>Prefer not to respond	Multiple choice	304	Participant Profiles and Relationship with the Rönne River
Q5	How often do you visit the river to carry out these activities?	Almost every day<br>A few times per week<br>A few times per month<br>Seasonally, warmer months<br>Seasonally, colder months<br>A few times per year<br>Do not know/prefer not to respond	Multiple choice	303	Participant Profiles and Relationship with the Rönne River
Q6	How much do you agree with the following statements? The river…	Provides a choice for learning about local and regional ecosystems<br>Is a place in nature I feel connected to<br>Provides me with restorative benefits/health benefits<br>Is beautiful<br>Is aesthetically important	Likert: 1. Strongly disagree 2. Disagree 3. Neither	302<br>301<br>300<br>302<br>302	Participant Profiles and Relationship with the Rönne River

ID	Question	Sentences	Scale	Responses	Theme
		Is an important part of my local identity<br>Is an important part of my local culture and traditions<br>Has spiritual significance to me (religious or non-religious)<br>Will sustain similar activities and benefits to people in the future as it does today	agree nor disagree<br>4. Agree<br>5. Strongly agree	301<br>301<br>301<br>301	
Q7	In your opinion, how has present hydropower and damming in the river affected the following?	Industrial use<br>Recreational activities (e.g., hiking, running, canoeing, fishing, snow fishing)<br>Industrial fishing<br>Tourism<br>Infrastructures<br>Residents' everyday well-being<br>Ecology/natural environment<br>Species richness (biological diversity)<br>Water pollution (e.g., temperature changes)<br>Hydromorphology (e.g., sediment transport, connectivity, riverbank development)<br>Cattle/animals<br>Fossil-free energy<br>Electricity prices<br>Climate (change)	Slider: -5= Very negative<br>0= Neither negative nor positive<br>5= Very positive	299<br>298<br>282<br>299<br>294<br>295<br>301<br>296<br>288<br>291<br>292<br>294<br>291<br>290	Hydropower and Restoration Opinions
Q8	How much do you agree with the following statements?	My ideal vacation spot would be a remote, wilderness area<br>I always think about how my actions affect the environment<br>My connection to nature and the environment is a part of my spirituality<br>I take notice of wildlife wherever I am<br>My relationship with nature is an important part of who I am<br>I feel very connected to all living things and the Earth	Likert: 1. Strongly disagree<br>2. Disagree<br>3. Neither agree nor disagree<br>4. Agree<br>5. Strongly agree	302<br>303<br>302<br>303<br>304<br>302	
Q9	Overall, do you think these changes will be positive or negative:	For you/your community<br>For your everyday activities<br>For your wellbeing<br>For the river in terms of water availability for households<br>For the river in terms of water availability for other purposes<br>For the river in terms of water pollution<br>In terms of the overall wellbeing of the municipality<br>For other species in and along the river	Slider: -5= Very negative<br>0= Neither negative nor positive<br>5= Very positive	302<br>299<br>299<br>292<br>296<br>293<br>298<br>296	Hydropower and Restoration Opinions
Q10	What type of activities would you like this area to offer in the future?	The area offers enough activities as of today<br>Recreational fishing<br>Subsistence fishing<br>Farming/animal grazing<br>Walking/running<br>Skiing<br>Other winter sport<br>Water sports (e.g., canoeing)<br>Snow scooter<br>Tourism<br>Birdwatching<br>Recreational nature activities (e.g., hiking, camping)<br>Aesthetics (e.g., photography, filmmaking, painting, visual arts)<br>Social or cultural activities (e.g., museums)<br>Water use (e.g., irrigation)<br>Hydropower<br>Other industry<br>Other<br>Prefer not to respond	Multiple choice	300	Participant Profiles and Relationship with the Rönne River
Q11	What is your favorite place by the Rönne River?	-	Map indicator	182	Participant Profiles and Relationship

ID	Question	Sentences	Scale	Responses	Theme
					with the Rönne River
Q12	Why is it your favorite place?	-	Free text, must be 20 characters or less	217	Participant Profiles and Relationship with the Rönne River
Q13	How certain can we be about the knowledge of consequences of the hydropower plant disassemble on …	Freshwater animals<br>Cultural values<br>Nature values<br>Agriculture<br>Fishing<br>Grazing animals	Likert: 1. Very uncertain 2. Quite uncertain 3. Neither uncertain nor certain 4. Quite certain 5. Very certain	292<br>295<br>296<br>294<br>295<br>294	
Q14	Are you engaged in/active in the management/development of the river in any way, and if so, how?	Not active<br>Political engagement<br>Environmental activism<br>Interest organization<br>Work<br>Landowner<br>Other<br>Prefer not to respond	Multiple choice	303	Opinions on Involvement in the Restoration
Q15	To what extent have you been …	Allowed to express your opinion/voice regarding the management of the river?<br>Allowed to influence the management and decisions of the river?	One choice: 1. Not at all 2. Little 3. A lot	302<br>300	Opinions on Involvement in the Restoration
Q16	Do you know of approaches that you could use today to participate or contribute to the development and management of the river?	Via local politicians<br>Consultation processes<br>Interest organizations<br>Social media<br>Petitions<br>Other<br>No/do not know	Multiple choice	304	Opinions on Involvement in the Restoration
Q17	In your opinion, what type of knowledge should the future planning of the river system primarily be guided by?	Technical knowledge<br>Economic knowledge<br>Political knowledge<br>Scientific knowledge<br>Local knowledge<br>Indigenous knowledge<br>Knowledge about landscape aesthetics	Ranking	299	Opinions on Involvement in the Restoration
Q18	State your opinion about the effects of hydropower	Has the hydropower in your local river had a negative or positive effect on biodiversity locally?<br>Has the hydropower in your local river had a negative or positive effect on society locally?<br>Has the hydropower in your local river had a negative or positive effect on you personally?	Slider: -5= Very negative 0= Neither negative nor positive 5= Very positive	296<br>299<br>294	Hydropower and Restoration Opinions
Q19	What species can currently be found in the river ecosystem?	Cod<br>Cockle<br>Eel<br>Blue mussel<br>Saithe<br>Salmon<br>Mackerel<br>Trout	Multiple choice	278	
Q20	What group of organisms are often used as freshwater indicators of high water quality?	Mussels<br>Aquatic insects<br>Invasive plant species<br>Terrestrial insects	Multiple choice	281	
Q21	What is your highest degree of education?	Elementary school<br>Upper secondary school	Single select	302	

ID	Question	Sentences	Scale	Resp onses	Theme
		Post-secondary education<br>College/university			
Q22	How old are you?	-	Free text, must be an integer between 0 and 120	304	Participant Profiles and Relationship with the Rönne River
Q23	Your gender?	Woman<br>Man<br>Non-binary<br>Transgender<br>Gender queer/gender fluid<br>Other<br>Prefer not to respond	Multiple choice	304	Participant Profiles and Relationship with the Rönne River
Q24	Do you want to participate in an interview about your relationship with Rönne å?	Yes<br>No	Single select	304	

Appendix S3 – Postcard used for distributing the questionnaire

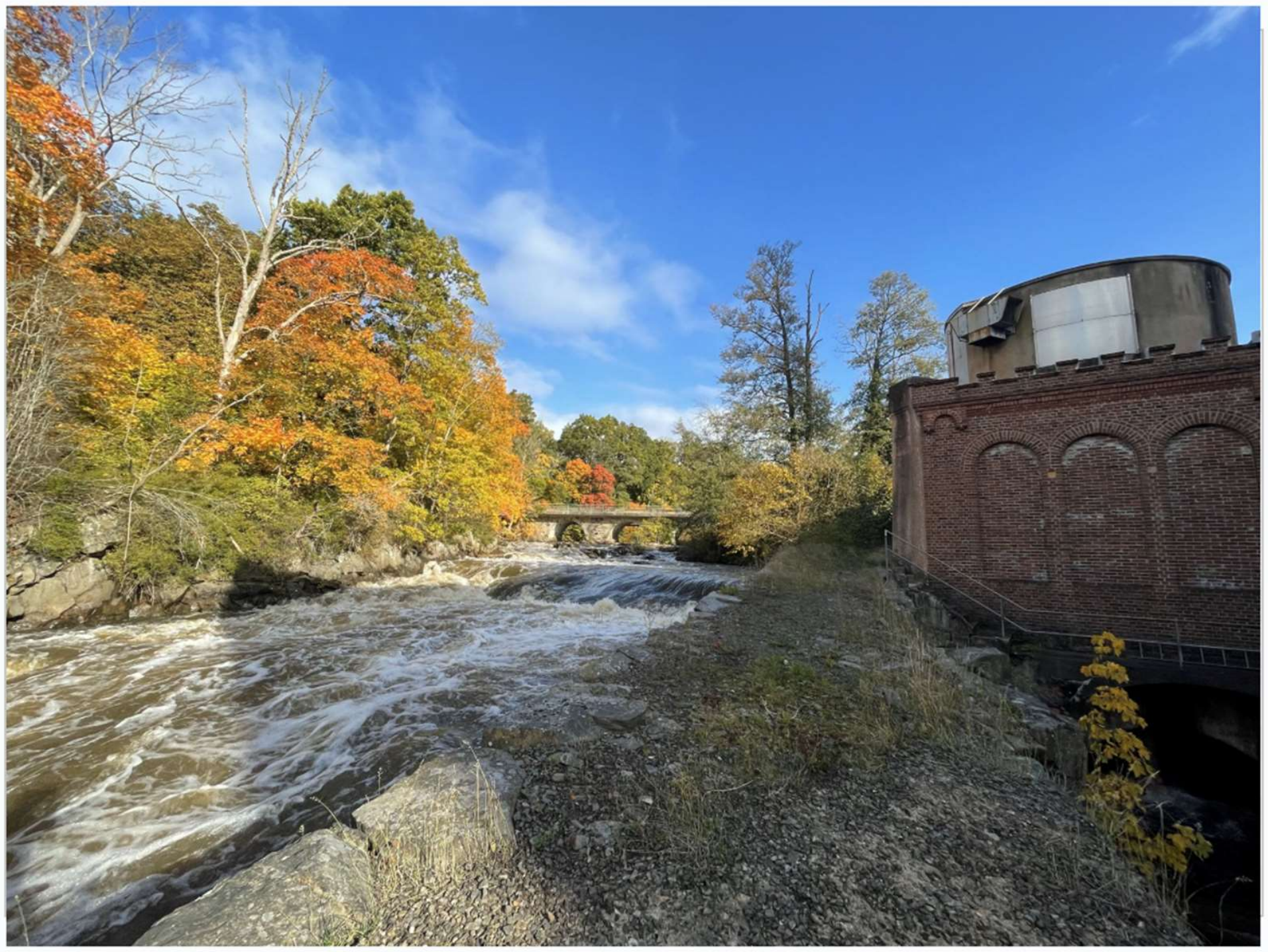

Sveriges Lantbruksuniversitet, Institutionen för Landskapsarkitektur, planering och förvaltning, Box 190, 234 22 Lomma

Vill du dela din åsikt om Rönne å?

Hur mår älven? Nu har du möjlighet att delta i en forskningsstudie som undersöker lokalbefolkningens aktiviteter och attityder kring Rönne å, vattenkraften och åns förvaltning. Vi är en grupp forskare som har tagit fram en enkät som tar ca 10 minuter att svara på. Dina svar är mycket värdefulla för hållbarhetsforskningen om Rönne å och av andra älvar i Sverige. Alla svar är anonyma.

Scanna QR-koden eller besök denna websida för att delta och se mer information:

arcg.is/1j15qf0

Ni kan även kontakta oss via mejl:

Scanna QR-koden med din mobilkamera

sanna.stalhammar@slu.se

SLU

Stockholm University

LUNDS UNIVERSITET

KARLSTADS UNIVERSITET

Appendix S4 – Information sheet for interviews

Information om att delta i studien "Restoring More Than Water: Exploring the Rönne River's Impact on People and Place"

Information om projektet och hur deltagarna valts ut

Jag är student på Masterprogrammet i globalisering, miljö och social förändring vid institutionen för naturgeografi vid Stockholms universitet. Jag vill fråga dig om du vill delta i en studie inom ett projekt som heter *Restoring More Than Water: Exploring the Rönne River's Impact on People and Place* (svenska: *Mer Än Vatten: Rönneåns Återställning och Dess Påverkan på Människor och Plats*). Genom detta projekt vill jag undersöka hur lokalinvånare uppfattar återställningen av Rönne å. Jag kommer att studera hur lokalinvånare uppfattar de landskapsförändingar och effekter som Rönne å-projektet medför. Dessutom kommer jag att studera hur lokalinvånare har involverats i planering och beslutsfattande för förvaltningen av Rönne å. Anledningen till att jag frågar just dig om du vill delta är att du är bosatt i närheten av Rönne å. Dessa uppgifter har jag fått tillgång till genom att du har anmält ditt intresse via den enkät du besvarade inom ramen för projektet. Mitt projekt ingår i ett större forskningsprojekt som heter *Putting locals first: mapping cultural values across rivers of the world* (svenska: *Med lokalbefolkningen i fokus: kartläggning av världens floders kulturella värden*) och leds av min handledare, Anna Scaini. Annas projekt kommer att utvärdera proxies för de sociokulturella tjänster som floder runt om i världen tillhandahåller, till exempel genom att kartlägga lokalbefolkningens favoritplatser vid floden. Forskningshuvudman för projektet är Stockholms universitet. Med forskningshuvudman menas den organisation som är ansvarig för projektet. Annas projekt är finansierat av Forskningsrådet för miljö, areella näringar och samhällsbyggande (Formas) (2022-00329), ett statligt forskningsråd för hållbar utveckling.

Vad det skulle innebära för dig att delta i studien

Om du tackar ja till att delta kommer det att innebära att jag genomför en individuell intervju med dig. Intervjun kommer att vara av semi-strukturerad karaktär vilket innebär att jag utgår från förutbestämda frågor som du kan besvara helt öppet, följt av eventuella följdfrågor som uppstår under intervjun. Intervjun beräknas ta omkring 60 minuter och hålls fysiskt eller via önskad digital mötesplattform på en tidpunkt som vi gemensamt kommer överens om. Hela intervjun kommer att spelas in för att underlätta min sammanställning. Det finns inga förutsedda risker för detta projekt men om du känner dig obekväm med att svara på någon utav frågorna kan du välja att avbryta intervjun när som helst utan negativa konsekvenser för dig och du behöver inte förklara varför.

Information om studiens resultat

Studiens resultat kommer att presenteras i en skriftlig uppsats. Insamlade data kommer att pseudonymiseras så när resultaten redovisas kommer det inte att framgå vilka personer som intervjuats. Jag kommer att delge dig den färdigställda uppsatsen och du kommer även kunna ta del av uppsatsen via Stockholms universitets publiceringsplattform för öppna data. Resultaten kan komma att publiceras i en tidskrift för vetenskapliga artiklar.

Det är frivilligt att delta

Det är helt frivilligt att delta i projektet. Du kan när som helst välja att inte vara med längre och du behöver inte säga varför. Om du väljer att inte längre vara med kommer detta inte att påverka relationen till Stockholms universitet. Om du inte längre vill vara med ska du meddela detta till den som ansvarar för projektet, se kontaktuppgifter nedan.

Behandling av personuppgifter

Om du väljer att delta kommer projektet att använda viss information om dig så som arbetsplats och dina intervjusvar. Denna information kommer att samlas in genom dina intervjusvar. Uppgifter som kan kopplas till dig på detta sätt räknas som personuppgifter enligt EU:s allmänna dataskyddsförordning 2016/679 (GDPR). Anledningen till att projektet behöver behandla sådana personuppgifter är för att kunna studera människors relation till och engagemang kring vattendrag.

Stockholms universitet är personuppgiftsansvarig för denna behandling. Den rättsliga grunden för personuppgiftsbehandlingen är allmänt intresse enligt EU:s dataskyddsförordning, artikel 6.1.

Personuppgifterna kommer att förvaras lokalt på en dator i Stockholm samt kopior sparas på Sunet Drive. För att projektet ska kunna utföras kommer vissa personer, jag som ansvarig för studien samt min handledare, att ges tillgång till personuppgifterna. Uppgifterna kommer att behandlas så att inte obehöriga kan ta del av dem.

När projektet är avslutat kommer data som samlats in och behandlats inom projektet att sparas i minst 10 år. Det sparade materialet kommer att innehålla pseudonymiserade personuppgifter. Om materialet bedöms ha ett bestående värde kommer det att bevaras för framtiden.

Enligt EU:s allmänna dataskyddsförordning samt nationell kompletterande lagstiftning har du rätt att:

- begära tillgång till dina personuppgifter
- få dina personuppgifter rättade
- få dina personuppgifter raderade
- få behandlingen av dina personuppgifter begränsad.

Under vissa omständigheter medger dataskyddsförordningen samt kompletterande nationell lagstiftning undantag från dessa rättigheter. Rätten till tillgång till sina uppgifter kan

exempelvis begränsas av sekretesskrav, och rätten att få uppgifter raderade kan begränsas av regler rörande arkivering.

Om du vill åberopa någon av dessa rättigheter ska du ta kontakt med projektansvarig, Emma Gudmundson, emmagudmundson99@gmail.com.

Om du har synpunkter på behandlingen av dina personuppgifter kan du kontakta dataskyddsombudet vid Stockholms universitet (dso@su.se) Integritetsskyddsmyndigheten (IMY) utövar tillsyn över behandling av personuppgifter i Sverige. Om du är missnöjd med hur dina personuppgifter behandlas har du rätt att klaga hos Integritetsskyddsmyndigheten. Information om detta finns på myndighetens webbplats (imy.se).

Kontaktuppgifter till ansvariga för projektet

Projektansvarig: Emma Gudmundson, tfn: XXX, emmagudmundson99@gmail.com.

Handledare: Anna Scaini, tfn: XXX, anna.scaini@natgeo.su.se.

Appendix S5 – Consent form for interviews

Samtycke till att delta i projektet “Restoring More Than Water: Exploring the Rönne River's Impact on People and Place”

Jag har läst och förstått den information om studien som anges i dokumentet ”Informationsblad”. Jag har fått möjlighet att ställa frågor och jag har fått dem besvarade. Jag får behålla den skriftliga informationen.

☐ Jag samtycker till att delta i studien som beskrivs i dokumentet “Informationsblad”

**Plats och datum**	**Underskrift och namnförtydligande**
....	....

Appendix S6 – Interview guide

Lära känna

1. Berätta lite om dig själv. Hur länge har du bott i området? Sysselsättning?

Din relation till Rönne å

2. Vad betyder Rönne å för dig?
    a. Vilken är din favoritplats vid Rönne å[1]?

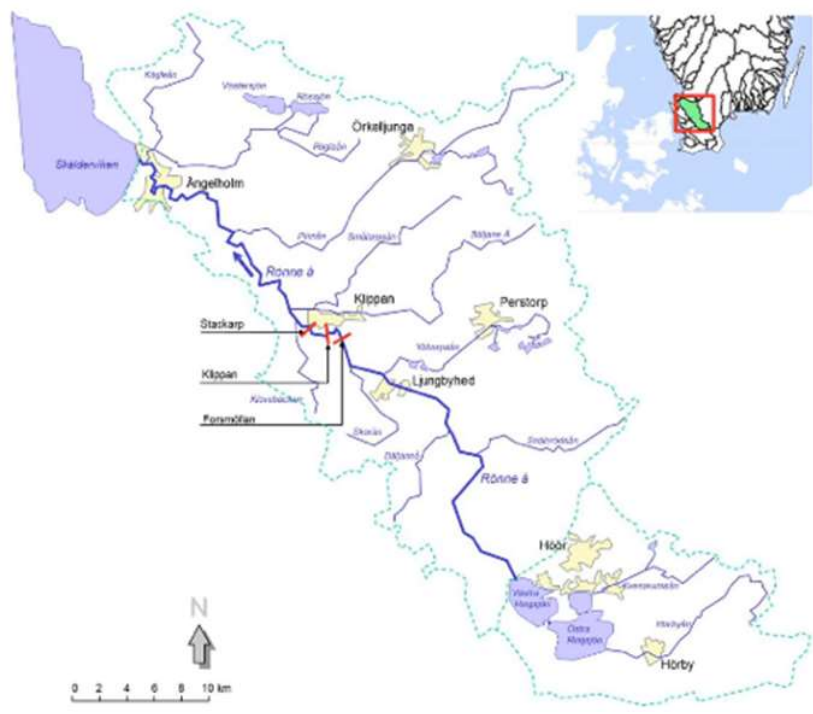

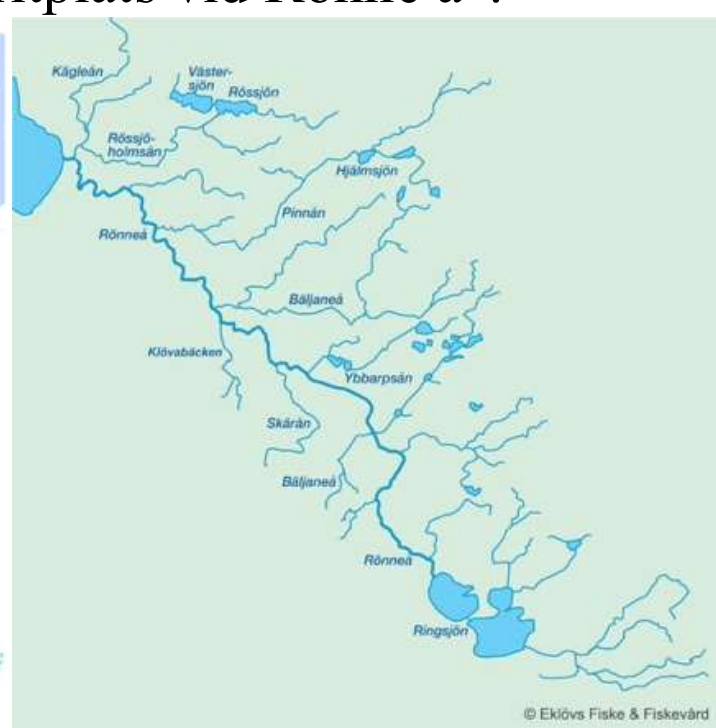

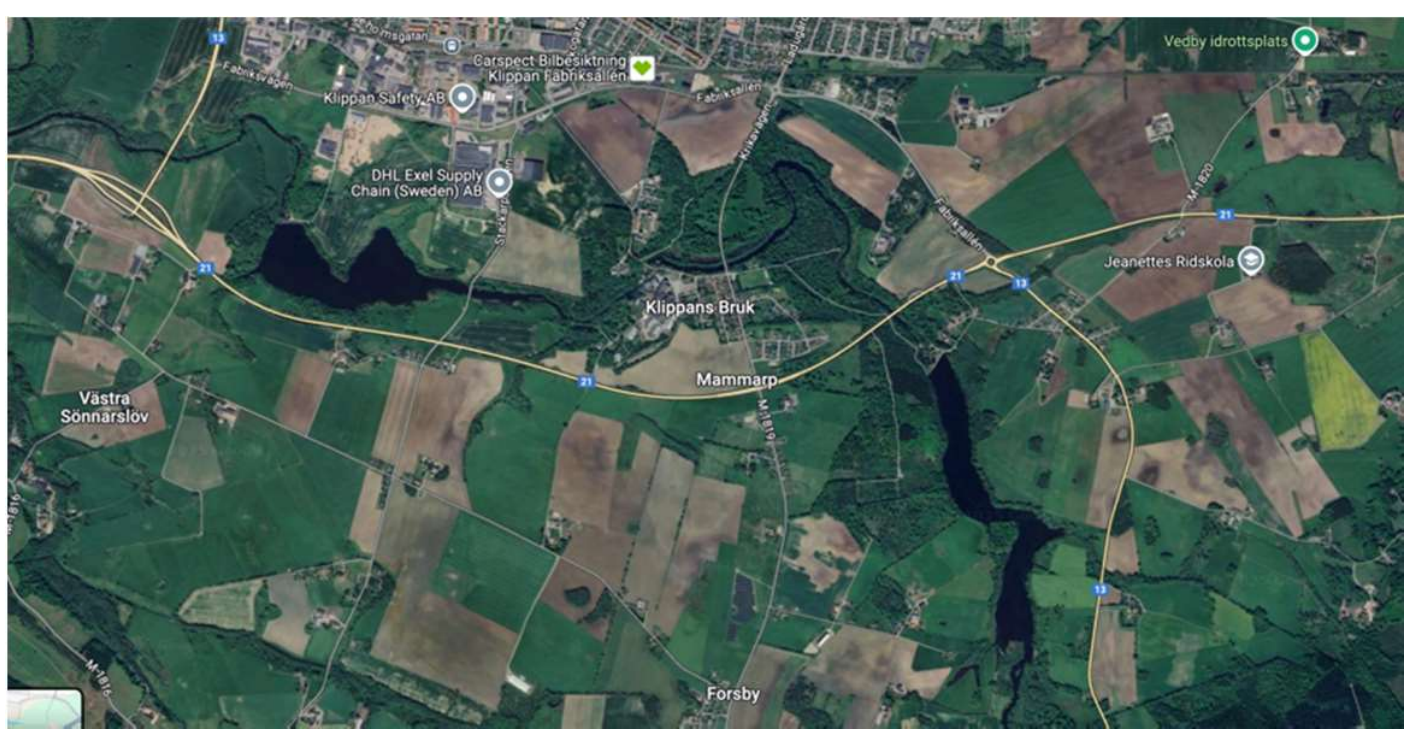

        i. Varför är denna plats din favorit?

Om Rönne å-projektet[2]

3. Hur har du fått kännedom om Rönne å-projektet?
4. Vad, enligt dig, är Rönne å-projektets främsta syften?
    a. Vad innebär en flodåterställning/restaurering för dig?
5. Vad är dina generella åsikter om Rönne å-projektet?
    a. Vilka aspekter tycker du får för lite uppmärksamhet i projektet?
    b. Har dina åsikter om projektet förändrats över tid?
    c. Hur uppfattar du andras åsikter om projektet?

Involvering i förvaltningen av Rönne å

6. Har du uttryckt dina åsikter om projektet offentligt?
7. Är du/har du varit involverad i projektet eller i annan förvaltning av Rönne å?

---

[1] *Under denna fråga kommer du att få interagera med kartor.*

[2] *I Rönne å-projektet har Klippans kommun köpt tre vattenkraftverk (Stackarp, Klippan bruket, Forsmöllan) för att nedmontera dem och återställa ån.*

a. Tycker du att det är enkelt eller svårt att engagera sig i förvaltningen av Rönne å?
b. Tycker du att förvaltningen av Rönne å baseras på rätt typ av kunskap idag?

Rönne å-projektets effekter/konsekvenser

8. Vad tror du kan vara möjliga konsekvenser av Rönne å-projektet?
   a. För dig som enskild invånare?
      i. Landskapsbildsförändringen[3]

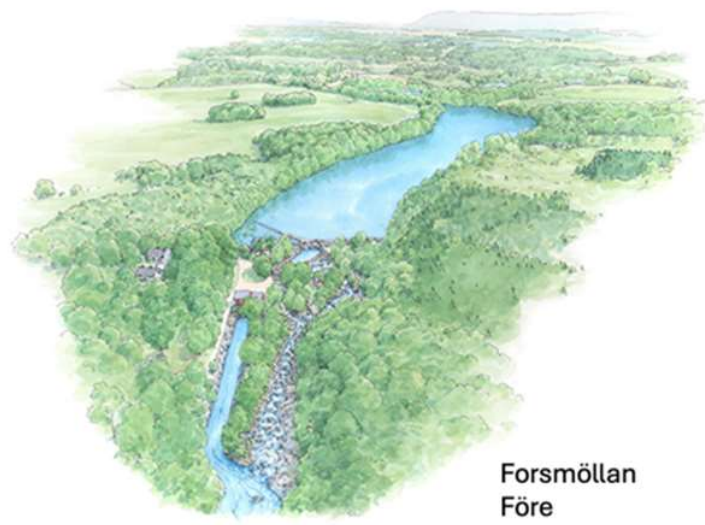

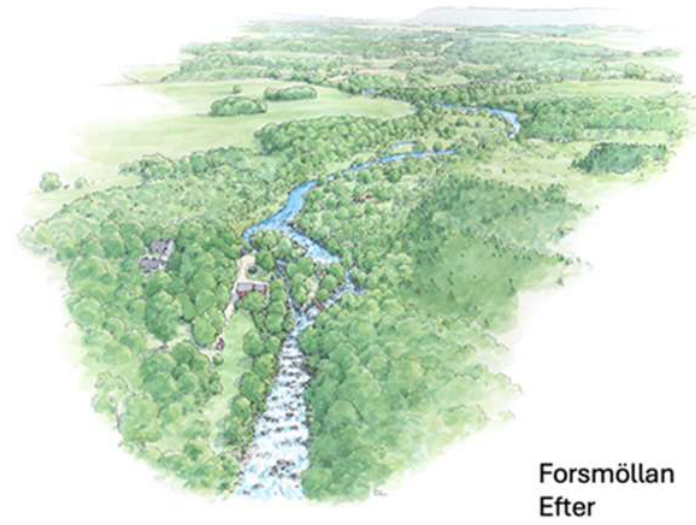

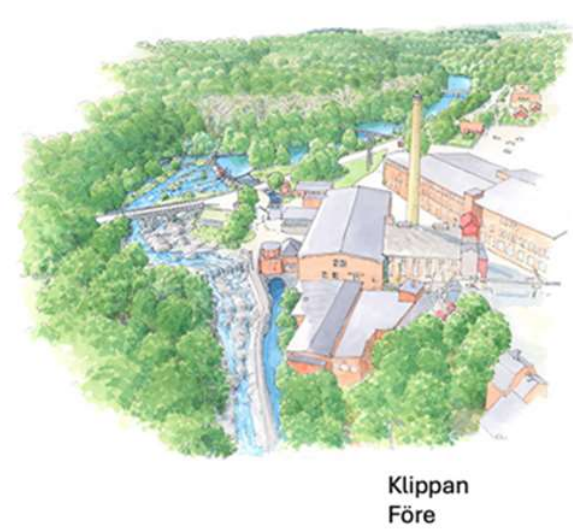

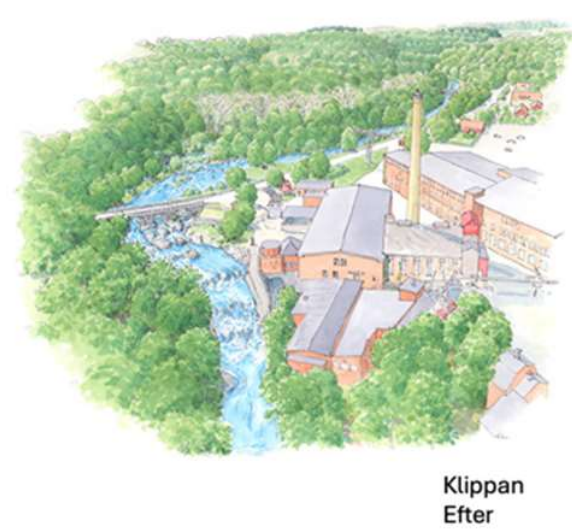

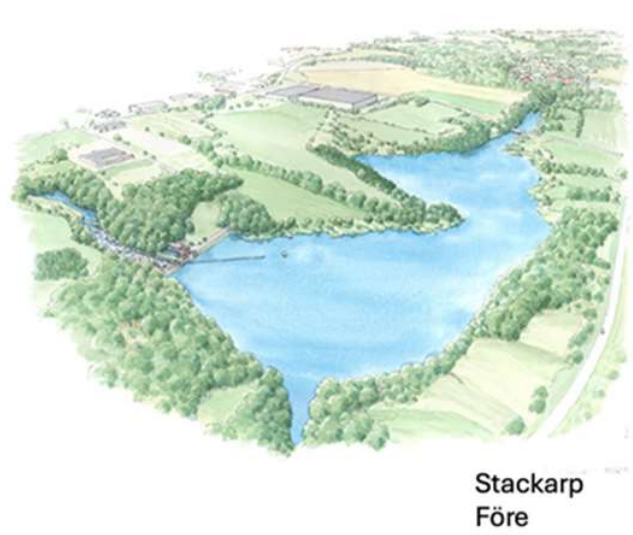

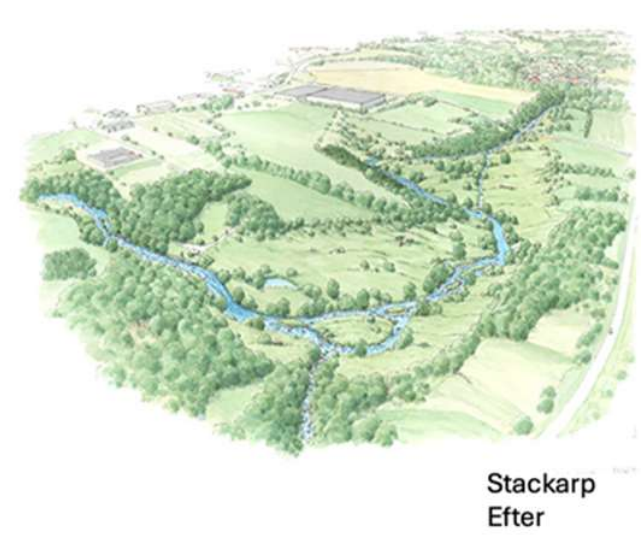

   a. För din hemkommun och de andra kommunerna kring Rönne å?
      ii. Ekologi, energi, kulturmiljö, rekreation, turism, landsbygdsutveckling
9. Vad tror du kan vara möjliga effekter av fler flodåterställningsprojekt i Sverige?

Avslutning

10. Har du några övriga frågor eller tillägg?

---

[3] *Under denna fråga kommer du få se före/efter-bilderna. Återställningen av Rönne å kommer att förändra landskapet i och i närheten av ån vid Stackarp, Klippan (bruket) och Forsmöllan.*

Appendix S7 – Additional questionnaire results

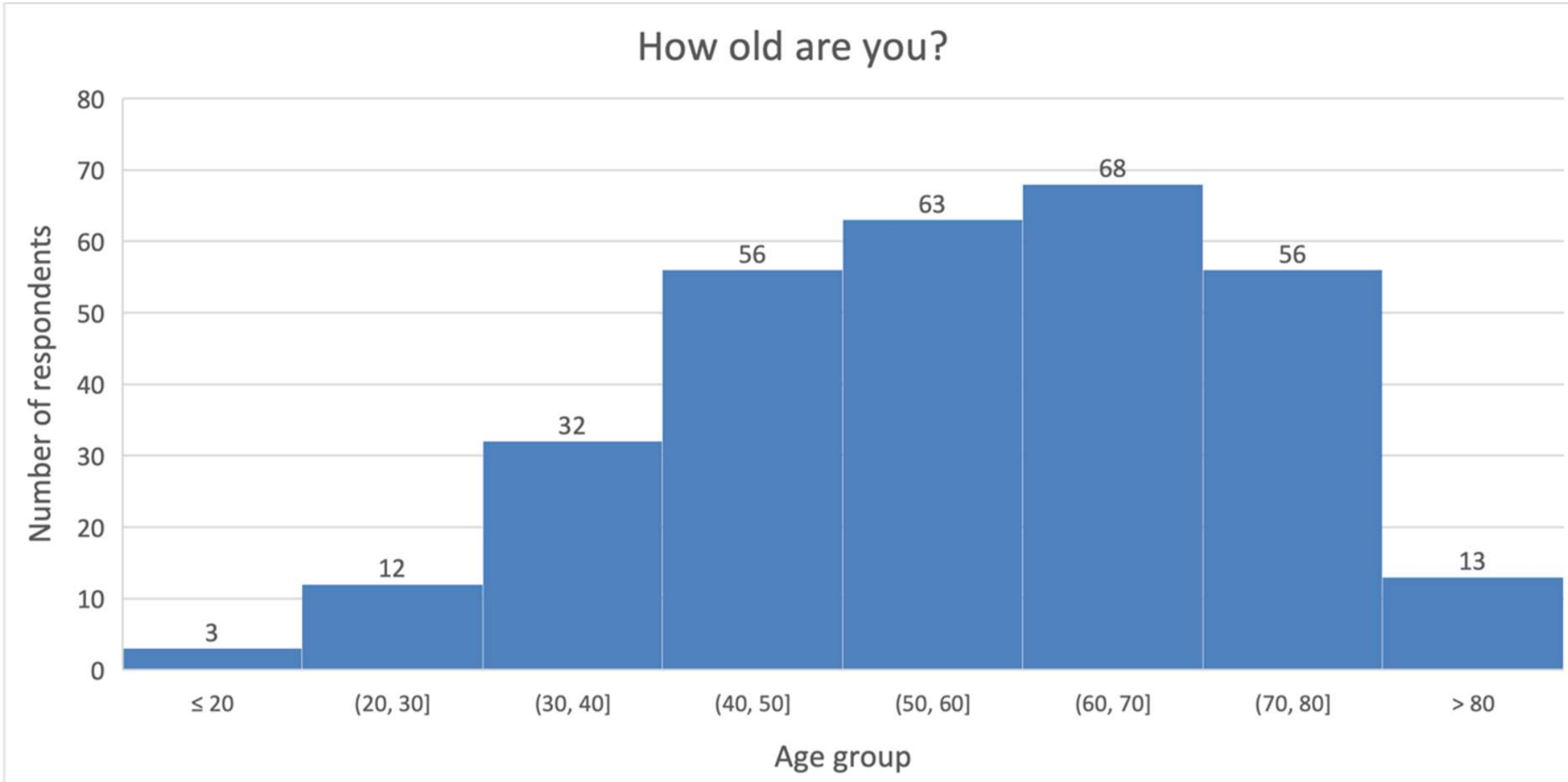

Figure S9: Frequency of questionnaire respondents across age groups.

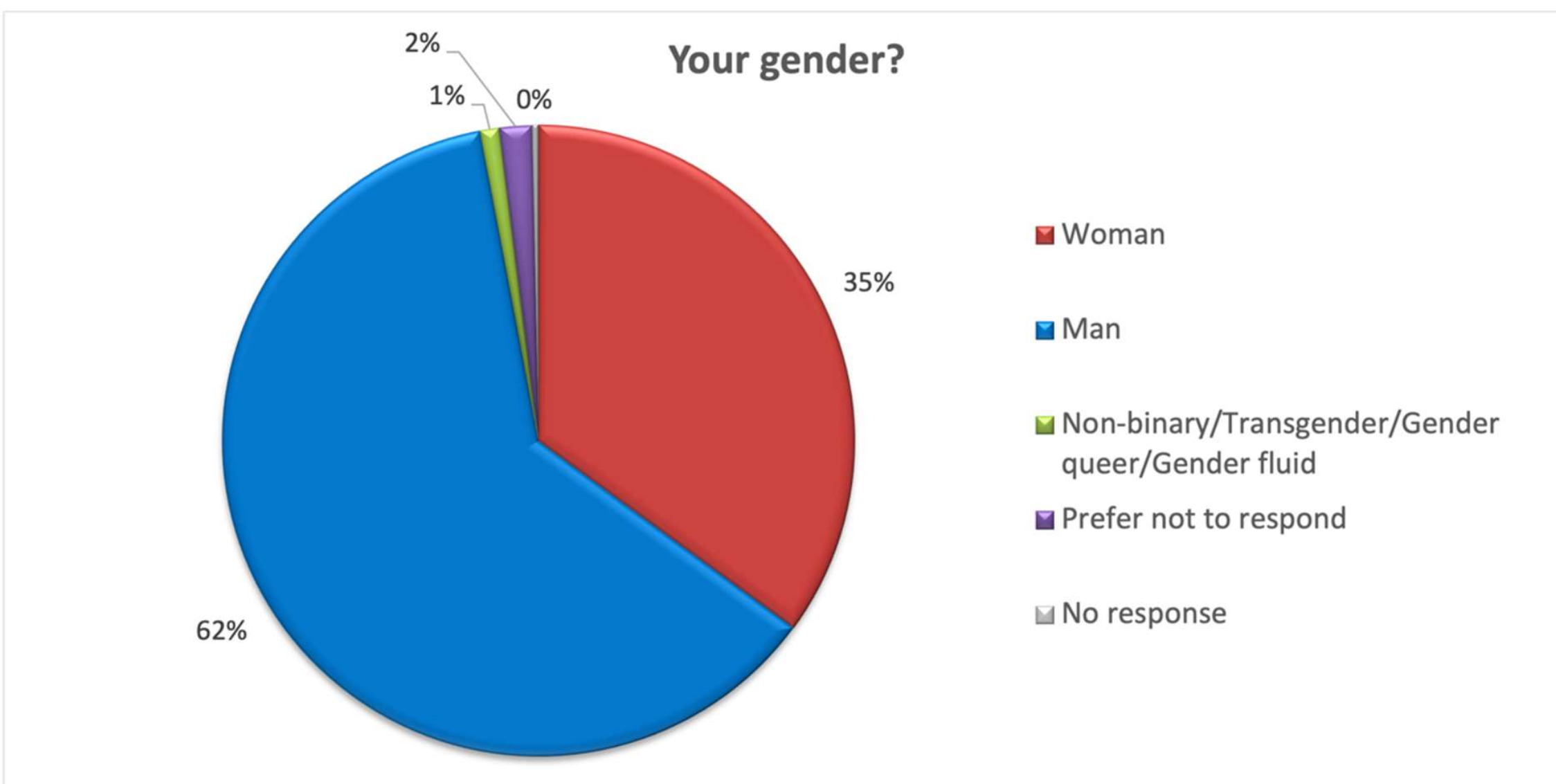

Figure S10: Distribution of questionnaire respondents by gender.

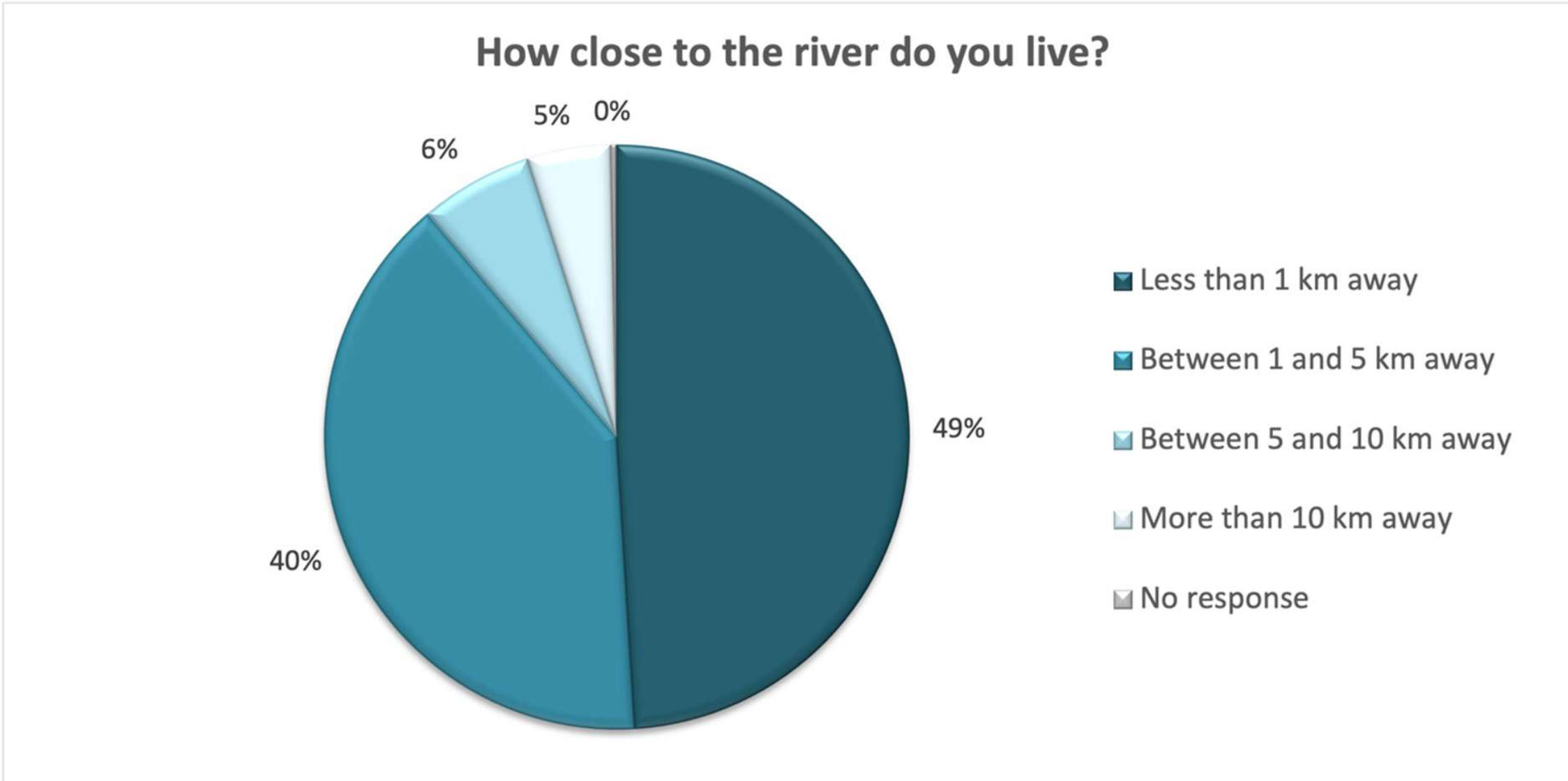

Figure S11: Residential proximity to the Rönne River among questionnaire respondents.

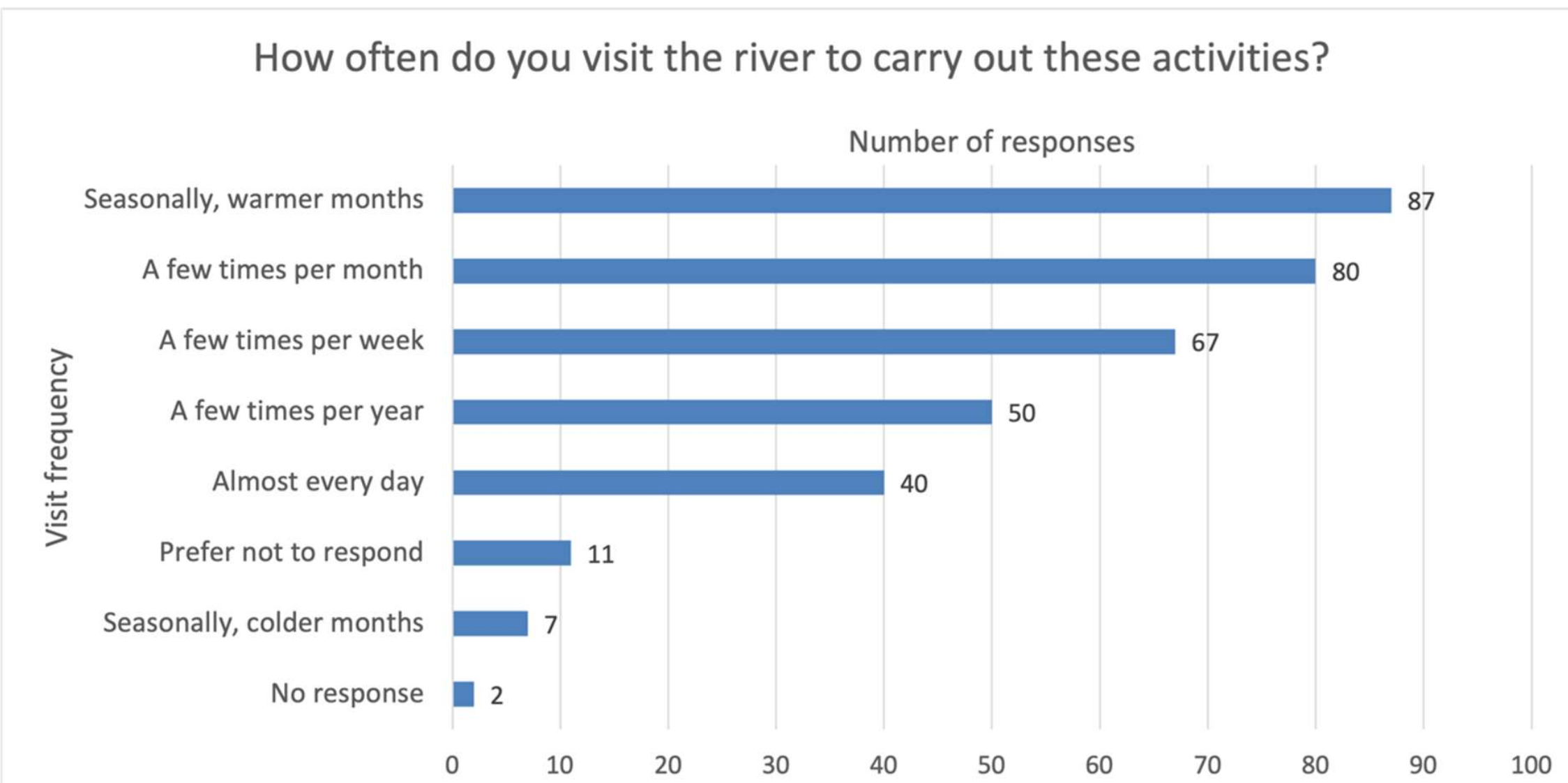

Figure S12: How often respondents visit the river to engage in the selected activities.

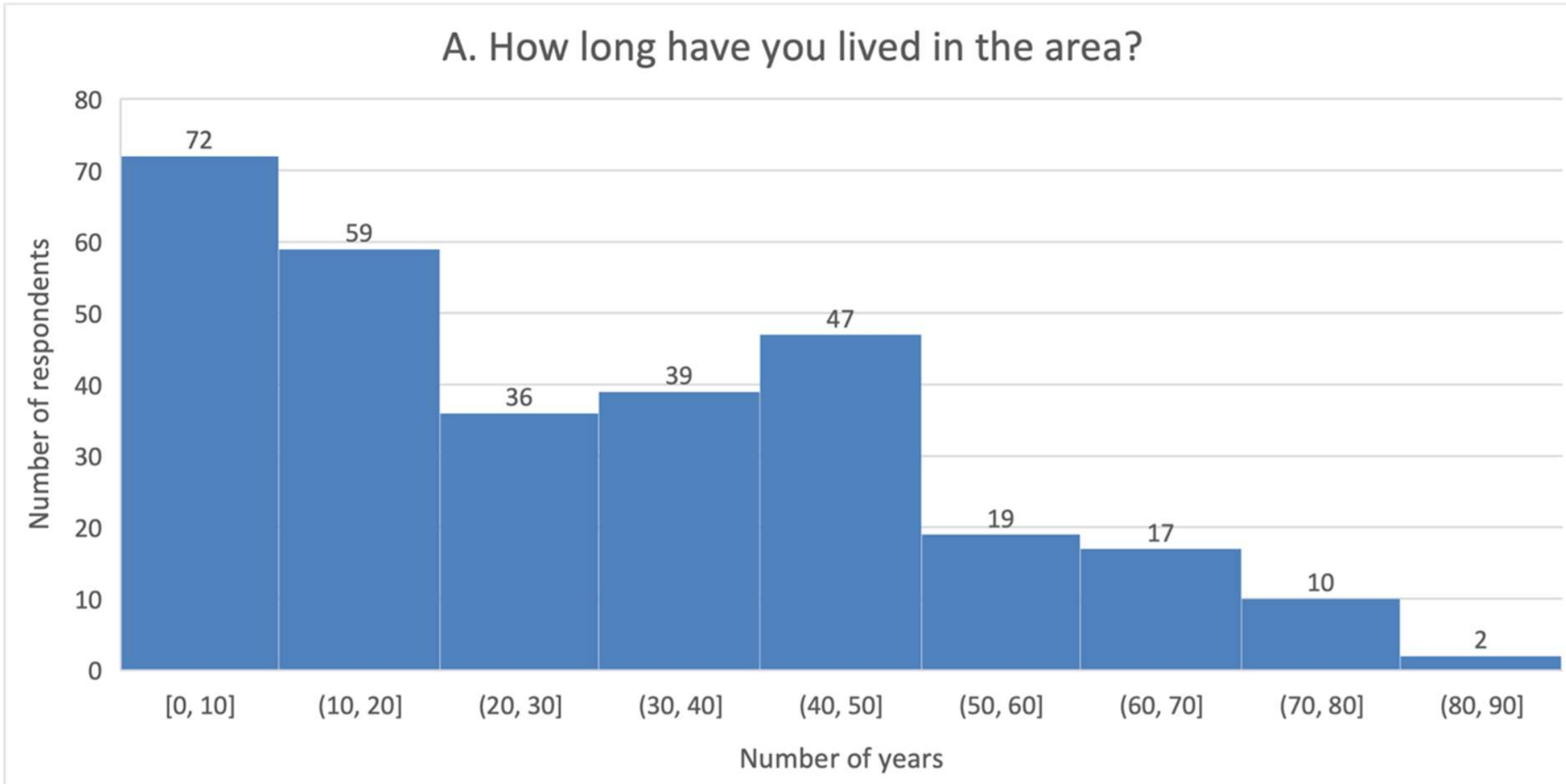

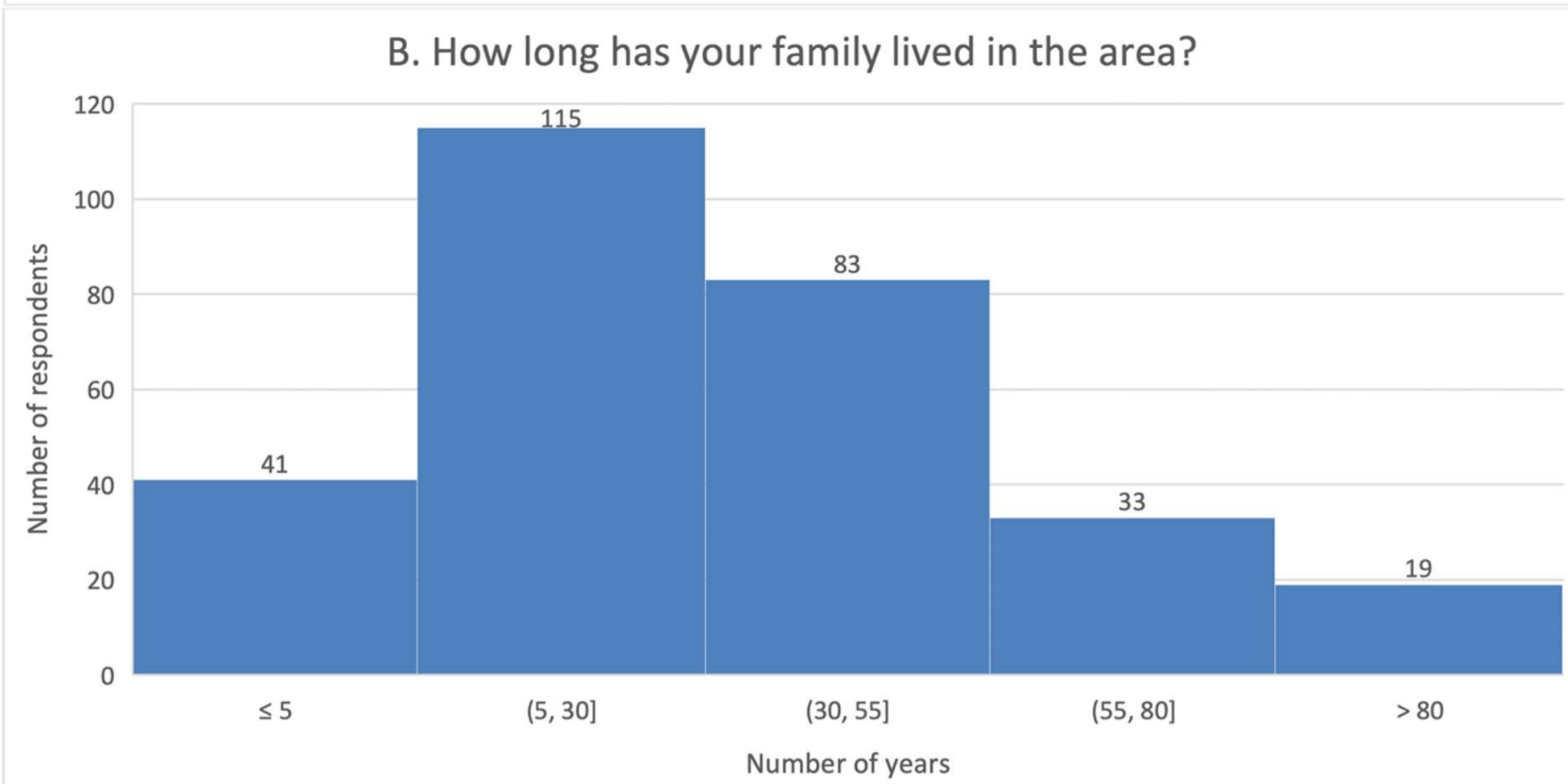

Figure S13: Distribution of questionnaire respondents by lenght of residence: A. Years the respondent has lived in the area; B. Years the respondent's family has lived in the area.

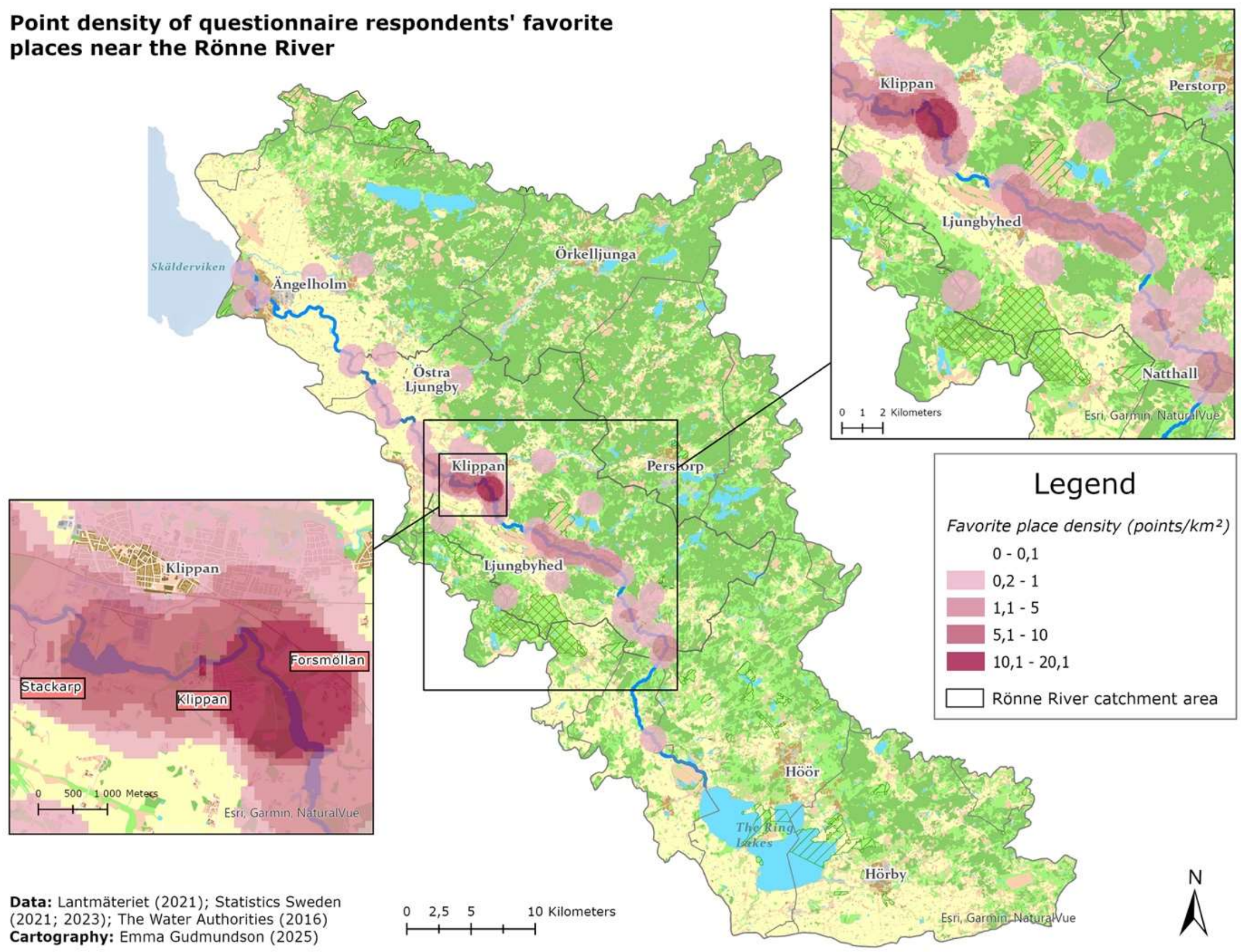

Figure S14: Map displaying point density of respondents' favorite places.

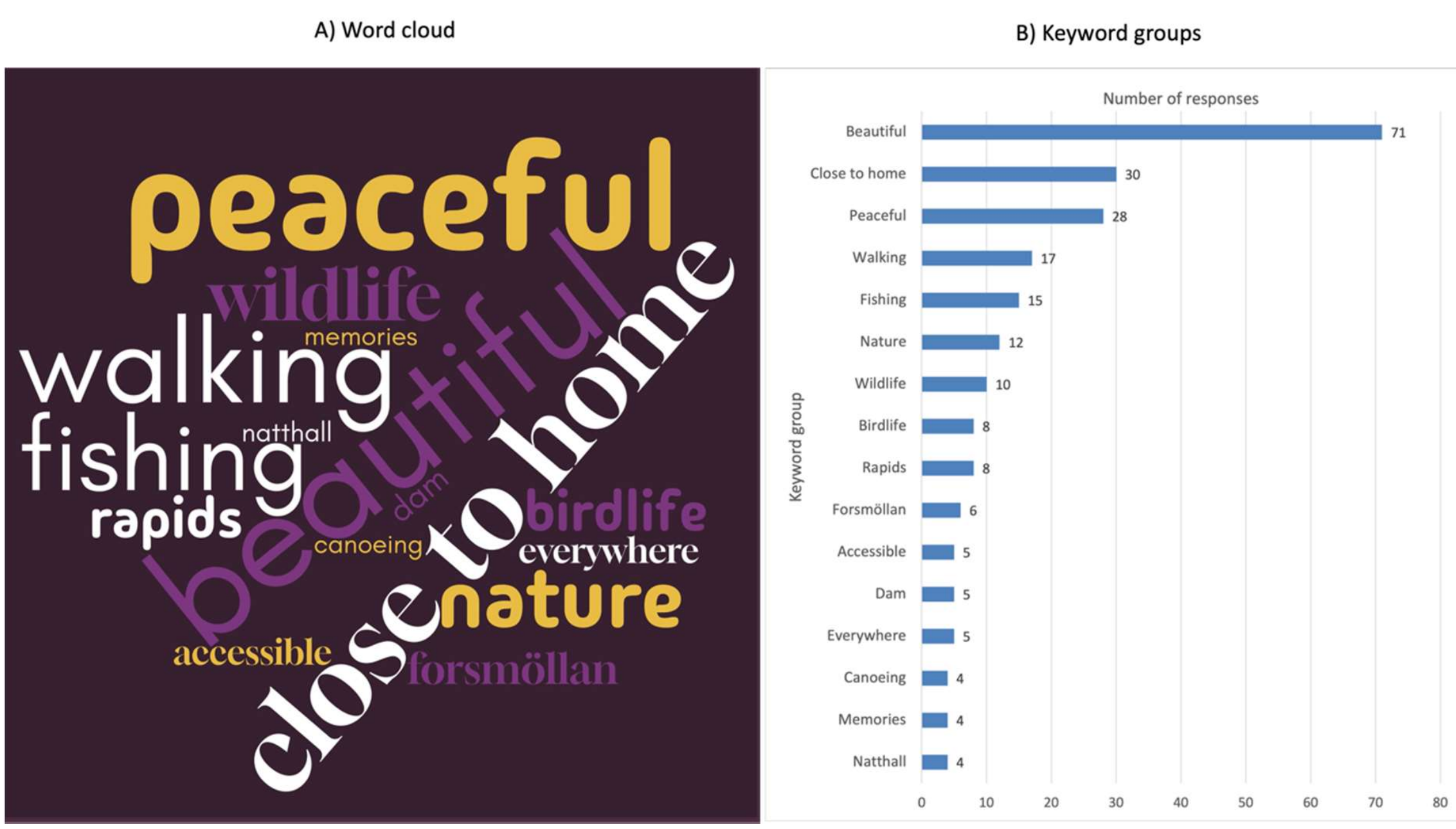

Figure S15: A. Word cloud displaying the 15 most common keyword groups extracted from free-text responses on reasons for choosing a locale as a favorite place. B. Frequency distribution of these 15 keyword groups.

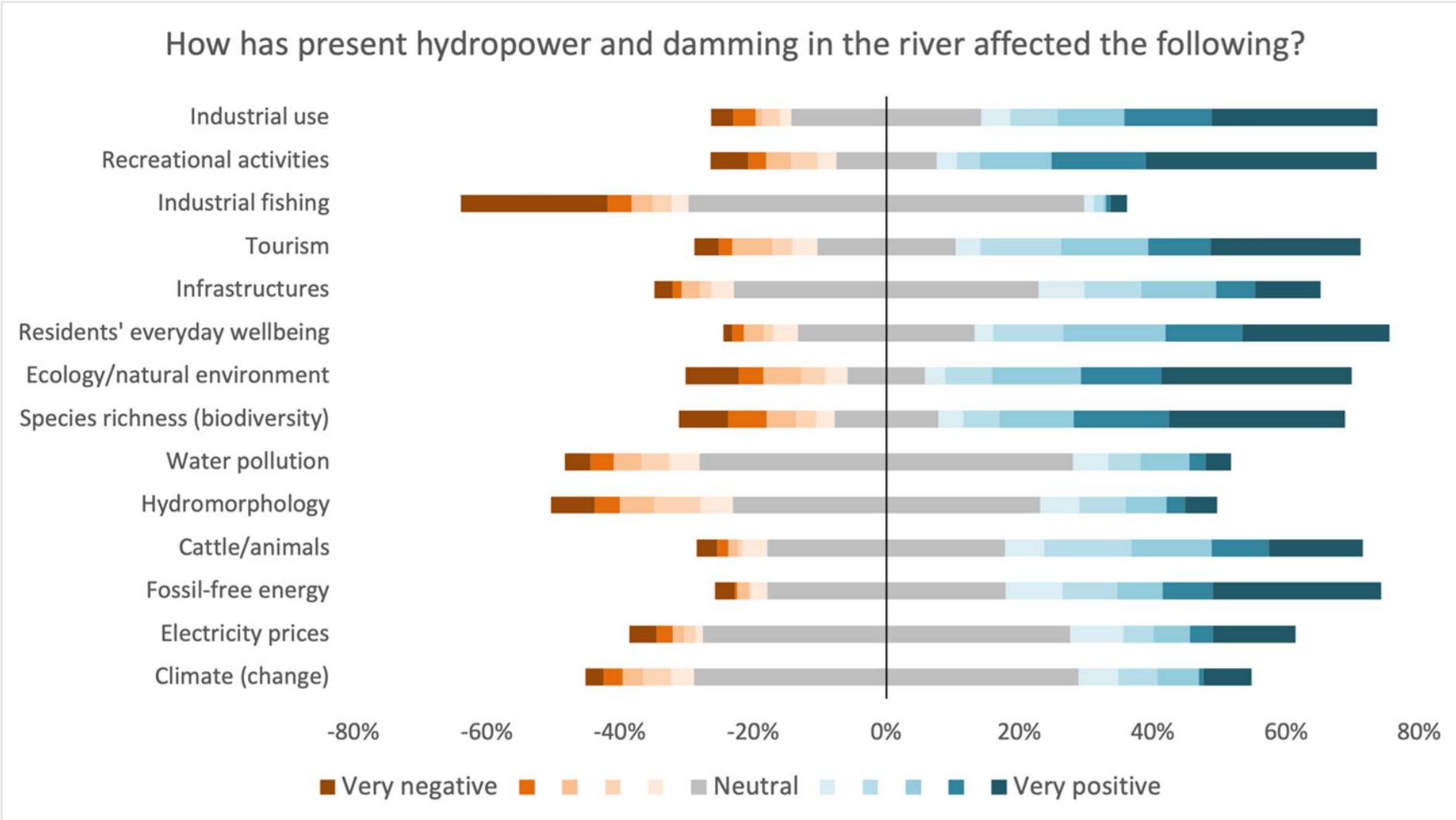

Figure S16: Respondents' opinions on the impact of current hydropower and damming in the Rönne River on various aspects.

Appendix S8 – Additional qualitative interview results

Participants' Relationships with the river Rönne å

The eight interviewees ranged from 30 to 83 years old, comprising six men and two women. Most lived in Klippan municipality, with one residing in Ängelholm municipality. Many had lived within walking distance of Rönne å. All reported visiting the river at least a few times per year for recreation and described strong personal connections to it. Interviewees agreed that repeated visits and shared memories have deepened these relationships over time. **As one explained, "*It has become more over the years. […]. At first, you do not have any relationships when you are a new resident, but they are built up over time*" (L1). Another interviewee highlighted a lifelong connection with flowing water in general, having always lived close to it.**

Some interviewees did not have a specific favorite place along the river but appreciated its overall aesthetics and recreational value. Others cherished spots tied to memories and emotions, especially those easily accessible from home. One interviewee highlighted sites that helped them symbolize life moments, for instance, describing a riverbank that occasionally floods: "*Just like life sometimes overflows with things that you must do. [...] it also becomes an image, that this is what it can look like, it can be flooded, but it then goes back to the normal course*" (L2). Beyond the tangible, this interviewee also emphasized rivers' roles as connectors of landscapes, forming natural networks like roads or nervous systems**: "*I think it is fantastic that the river connects different places in the country. […] That here there is a natural path in the landscape. [...] Even if you look at a map, it is like a nervous system in that everything is connected*" (L2).**

One questionnaire respondent echoed this perspective, emphasizing enjoyment derived from observing nature rather than actively using its resources, and highlighting the importance of recognizing the rights of non-human species. They suggested that humans might not automatically have rights to all parts of the river and that human interactions – both before and after restoration – shape the well-being of the entire river ecosystem (R2). Similarly, an interviewee argued that the river itself should be given a voice in decision-making, stressing that municipalities and their partners ought to act as its representatives: "*That the river has a say, that it cannot just be used for hydropower*" (L2).

Perceptions of Hydropower and the Restoration

Most interviewees expressed support for the restoration project, while others were critical. Interviewees generally spoke optimistically about the ecological goals, highlighting benefits for wildlife in and around the river. Reflecting on the trade-offs, one interviewee noted that "*those power plants did not produce that much electricity, but hopefully they can provide greater benefits for nature", emphasizing that "for biodiversity, [...] it is something I think is very important, because in the long run, our lives also depend on it*" (L6). Supporters valued knowing that the ecosystem would function more effectively, even if they could not always describe the exact impacts and acknowledged that biodiversity benefits would not always be directly perceptible but still appreciated the long-term ecological focus of removing the dams.

A recurring critical perspective was that closing functioning hydropower plants is an unwise environmental and political choice. However, most interviewees agreed on the three hydropower plants' insignificant effect on the electricity system. One interviewee suggested that much local skepticism reflects a general resistance to change and mistrust of political institutions rather than specific objections to the restoration itself:

> ***They just do not want anything to change. [...] If it had been 70 years ago, they would never have wanted to build any power plants [...] There is some kind of suspicion and lack of trust in politicians and other people in power. [...] That it is now about power plants, it could have been about a factory or a school or a road, wind turbines.*** **(L8)**

Drawing parallels with previous local projects, the interviewee predicted that acceptance would grow once the project's benefits become visible, adding that:

> *Inside Klippan, where they have restored the leather factory [...] We who grew up in Klippan, the leather factory was central. I do not think a single person is sad that it is gone, and they have tried to restore a piece of land and make it available to the citizens. I think the same thing will happen with the power plants; in time, people will think it is good.* (L8)

Interviewees expressed mixed views on anticipated landscape changes. **In Stackarp, several noted that the changes would be particularly dramatic.** Some valued the current dam landscapes, appreciating tranquil areas like Forsmöllan, with one questionnaire respondent noting that the existing landscape has shaped the upbringing of at least three generations (R1). **Others favored a more natural, free-flowing river and welcomed the transformation. Many expressed interests in observing the regrowth of plants and animals, considering it beneficial for biodiversity and recreation.** Some described the dams as more of a hindrance than an aesthetic feature, recalling canoeing through the Forsmöllan dam, where paddling rattles against submerged trees: "*It looks like a forest underwater, actually. Then you think about it even more, that it was not like that once*" (L3).

The hydropower plants are widely regarded as part of the area's historical and cultural heritage, having contributed to the local community for centuries. For some, the restoration itself also represents as form of cultural development. One interviewee remarked, "*in a way, this also becomes a cultural landscape because it is we humans who are doing this – the restoration is also by human hands*" (L8). Similarly, another interviewee drew parallels to past modifications of another river, suggesting that altering the landscape can itself preserve history: "*The history remains. It is almost culture because it was redirected. I cannot imagine that it would be negative to just redirect the water, because you can still tell the story about the dam*" (L7).

Several interviewees suggested that raising awareness of the river Rönne å restoration could enhance environmental reflection among locals. One interviewee emphasized that such projects can subtly shape environmental thinking, explaining that initiatives like these may "*train ourselves to think in a certain way*" (L8) and influence broader environmental behavior.
